# Regenerative Outcomes of Combining siCOL1A2 Hydrogel with Acupuncture in a Rat Model of Chronic Intervertebral Disc Degeneration

**DOI:** 10.3390/bioengineering11111066

**Published:** 2024-10-25

**Authors:** Qianfu Zhang, Zhixuan Li, Sihan Zhou, Ji Li

**Affiliations:** 1 Department of Integrated Traditional and Western Medicine, Huashan Hospital, Fudan University, Shanghai 200040, China; zqf920115@163.com (Q.Z.); sihanzhou2008@163.com (S.Z.); 2 Technical Management Department, IIJ Global Solutions China Inc., Shanghai 200031, China; 15000203305@163.com

**Keywords:** intervertebral disc degeneration, cryogel, inflammation, siCOL1A2, acupuncture, chronic pain

## Abstract

Intervertebral disc degeneration (IVDD) is a significant cause of chronic pain and disability, necessitating innovative therapeutic strategies. This study investigates the combined effect of a novel siCOL1A2-encapsulated hydrogel and acupuncture on IVDD in a rat model. We developed a hydrogel system, siCOL1A2-encapsulated G5-PBA hydrogel (siCOL1A2@G5-PBA@Gel), designed for sustained siRNA delivery to the degenerated discs and assessed its therapeutic efficacy alongside acupuncture treatment. Key inflammatory genes were identified through RNA-seq analysis, with COL1A2 highlighted as a crucial regulator of inflammatory responses in IVDD. Our in vivo experiments involved treating rats with hydrogel alone, acupuncture alone, and combining both. The treatments were evaluated through behavioral pain assessments, imaging techniques (X-ray and MRI), and histological analyses. Results indicated that the combination therapy significantly alleviated pain, reduced inflammation, and promoted disc regeneration more effectively than individual treatments. The hydrogel proved biocompatible and facilitated targeted gene silencing, while acupuncture enhanced therapeutic outcomes by improving local blood circulation and modulating inflammatory responses. These findings suggest that integrating siCOL1A2 hydrogel with acupuncture offers a promising approach to treating IVDD.

## 1. Introduction

Intervertebral disc degeneration (IVDD) is considered a common cause of lower back pain and leg pain and is recognized as a degenerative disease of the spine. The deterioration of the intervertebral discs is typically the cause of this condition, wherein the herniation of the nucleus pulposus due to the partial or complete rupture of the annulus fibrosus can irritate or compress the nerve roots and cauda equina, leading to pain and other symptoms [1]. The occurrence of IVDD is closely associated with factors such as inflammatory responses, cell apoptosis, imbalanced collagen synthesis, and changes in the extracellular matrix (ECM) [2,3,4,5].

Current treatment methods primarily include medication, physical therapy, and surgical intervention, necessitating close monitoring of patients post-repair to observe signs such as bleeding, swelling, and discharge at the surgical site; assessing lower limb muscle strength, sensation, and mobility; preventing possible postoperative complications like nerve root edema and disc space infections; and conducting radiographic follow-ups of the disc tissue in the short term [6]. However, these approaches pose certain side effects and risks, such as cardiovascular damage, myocardial infarction, and cartilage loss [7], with limited effectiveness in symptom improvement and disease progression prevention [8,9,10]. Therefore, the development of novel treatments for IVDD holds significant clinical implications.

Cryogenic hydrogel and hydrogel materials are under extensive research and application in the treatment field due to their excellent biocompatibility and sustained release properties [11,12]. Studies have explored the combination of hydrogels with drugs or bioactive substances for IVDD treatment [13,14,15,16,17]. Recent research also suggests that hydrogel carriers combined with gene therapy can more effectively alleviate IVDD symptoms [18]. Acupuncture therapy, with a long history in traditional Chinese medicine clinical practice, has been proven to significantly relieve pain and inflammation [11]. Acupuncture treatment works by improving blood circulation, reducing pain stimuli, and promoting tissue repair to alleviate pain and improve function [19,20,21]. Studies indicate that acupuncture treatment is beneficial for chronic pain in IVDD patients [19,20,21]. Therefore, acupuncture therapy shows promising potential in the treatment of IVDD. Given this existing knowledge, the potential application of hydrogel carriers delivering gene therapy in combination with acupuncture treatment for IVDD warrants further investigation.

This study aimed to investigate the therapeutic effects and mechanisms of cryogel/hydrogel combined with acupuncture therapy on chronic pain in a rat model of IVDD. First, IVDD-related RNA-seq chips in the gene expression omnibus (GEO) database were analyzed to identify key genes associated with inflammation. Next, a rat model of IVDD was established and divided into different treatment groups for intervention experiments [22,23,24]. In addition, a gene delivery vector, G5-PBA, was constructed to stably bind and silence the COL1A2 gene, which was then embedded in hydrogel to form siCOL1A2@G5-PBA@Gel. Finally, the effects of siCOL1A2@G5-PBA@Gel combined with acupuncture therapy on promoting nucleus pulposus (NP) regeneration, as well as its impact on pain response, IVDD, expression of inflammatory factors, and cell apoptosis, were evaluated through the rat model experiment [25].

IVDD severely affects patients’ quality of life, making the search for more effective treatment methods of great clinical significance. The findings of this study can provide new treatment options for patients with IVDD and contribute to the understanding of the occurrence and development mechanisms of this disease. Ultimately, through this study, we hope to provide effective treatment methods for IVDD, improve patient symptoms, and provide a scientific basis for clinical treatment and rehabilitation.

## 2. Materials and Methods

### 2.1. Animal Ethics Statement

All animal experiments in this study were conducted in strict accordance with the International Guidelines for Animal Ethics and Welfare and have been approved by the Animal Ethics Committee of our research institution. Throughout the experimental process, we adhered rigorously to the principles of minimizing animal pain and stress to ensure animal welfare. All experimental procedures are carried out by trained personnel under strict control conditions. Furthermore, our research complies with relevant national and regional laws and regulations to ensure the legality and ethics of scientific research.

### 2.2. Data Downloading and Analysis of GEO Microarray

The IVDD expression profile dataset GSE15227 was obtained from the Gene Expression Omnibus (GEO) database (https://www.ncbi.nlm.nih.gov/gds) (accessed on 15 May 2023). This dataset was generated using the GPL1352 sequencing platform and includes three unhealthy (grade four) intervertebral disc tissues (GSM380296, GSM380299, GSM380308) and twelve healthy (grades two and three) intervertebral disc tissues (GSM380295, GSM380297, GSM380298, GSM380300, GSM380301, GSM380302, GSM380303, GSM380304, GSM380305, GSM380306, GSM380307, GSM380309). The R package “limma” was applied to filter differentially expressed genes (DEGs) using the criteria |logFC| > 1 and a corrected *p*-value < 0.05. All analyses were performed using R version 4.3.1 (R Foundation for Statistical Computing).

### 2.3. Selection of Candidate Targets

Target genes related to IVDD and inflammation were retrieved from the GeneCards database (https://www.genecards.org/, accessed on 15 May 2023). The terms “Intervertebral Disc Degeneration” and “inflammation”, were searched to obtain a list of relevant genes, and the filtering criteria of the GeneCards database were set to a Relevance Score > 1 to obtain more relevant targets for subsequent analysis. Candidate targets were identified through Venn analysis by combining IVDD-related target genes, inflammation-related genes, and DEGs from the GEO microarray analysis [26,27].

### 2.4. GO and KEGG Functional Enrichment Analysis

To assess the functionality of candidate targets in IVDD, the clusterProfiler package in R software was employed to conduct gene ontology (GO) and Kyoto Encyclopedia of Genes and Genomes (KEGG) pathway enrichment analysis on co-expressed genes. The GO enrichment analysis included biological processes (BP), molecular functions (MF), and cellular components (CC). The candidate targets were ranked based on their *p*-values and subjected to enrichment analysis using online tools. The obtained data was visualized using the ggplot2 package [26].

### 2.5. PPI

By inputting the genes encoding the candidate targets’ proteins into the STRING database, protein-protein interaction (PPI) information was retrieved. Cytoscape V3.9.1 software was used to visualize and rank the PPI network using the Degree sorting method. Furthermore, the top 10 genes based on their Degree ranking were calculated and visualized using CytoHubba V3.9.1 [28].

### 2.6. LASSO Regression and Random Forest Analysis

LASSO Regression (Least Absolute Shrinkage and Selection Operator regression) was employed for variable selection and regularization in regression models. The optimal regularization parameter (λ) was determined through cross-validation, which involved fitting the model across a range of λ values and selecting the one that offered the best performance. At the chosen λ value, the coefficients were examined, with LASSO Regression typically shrinking some coefficients to zero, thus aiding in variable selection. The analysis was conducted using the “glmnet” package in R software [29]. Random Forest (RF), a supervised machine learning algorithm based on decision trees, was extensively applied to both regression and classification tasks. This method improves prediction accuracy and robustness by aggregating the outputs of multiple decision trees. In Random Forest, each tree independently learns from the data, and the combined results form the final prediction [30]. By integrating these two methods, we minimized the impact of arbitrary selection, enhancing the reliability and biological relevance of the identified hub genes. This approach was instrumental in pinpointing genes truly associated with IVDD, providing strong molecular candidates for biomarker discovery and research into pathological mechanisms.

### 2.7. Visualization of ROC Curves and Differential Expression

The receiver operating characteristic (ROC) curve was generated using the pROC package in R to evaluate the accuracy of predicting disease status based on gene expression. Both training and validation datasets were used, with gene expression values from candidate genes. Additionally, the expression levels of hub genes in the control and IVDD patient groups were visualized using box plots created with the ggplot2 package in R. A significance level of *p* < 0.05 was used to indicate statistically significant differences.

### 2.8. Pearson Correlation Analysis

To obtain a list of genes associated with apoptosis, aging, and inflammation, the GeneCards database (https://www.genecards.org/, accessed on 15 May 2023) was utilized. The search terms “Apoptosis”, “Aging”, and “Inflammation” were employed. The top 10 genes showing the strongest correlation with other genes were selected, and their correlations were calculated using the Pearson statistical method.

### 2.9. Isolation and Processing of NP Cells

The experiments involved three male Sprague-Dawley rats (270–300 g, catalog number 101, Beijing Vetonlihua Experimental Animal Technology Co., Ltd., Beijing, China). Under sterile conditions, the skin, muscles, and other soft tissues were removed from the rats’ lower back and tail to extract the intervertebral disc tissue. The tissue was then washed multiple times in PBS to remove blood and impurities. The NP tissue was isolated using microsurgical instruments, cut into pieces, and placed in DMEM/F12 medium (catalog number: 11320033, Thermo Fisher, Waltham, MA, USA). It was transferred to a 15 mL centrifuge tube and centrifuged at 1000 rpm for 5 min to remove the supernatant while keeping the sediment. The tissue was then washed three times with PBS. The tissue was minced and digested in a digestion solution containing 0.25% trypsin and EDTA and incubated at 37 °C for 30 min with occasional agitation to separate the cells. After digestion, the cells were collected by centrifugation at 1000 rpm for 5 min, the supernatant was removed, and an appropriate amount of PBS was added for three repeated washes. Afterward, the cells were digested again in DMEM/F12 medium containing 0.1% type 2 collagenase at 37 °C for 4 h, followed by three washes with PBS. Finally, the cells were seeded in suitable culture plates and cultured in DMEM/F12 complete medium containing 10% FBS (catalog number: 10100147C, Thermo Fisher, USA) and 1% antibiotics (100 U/mL penicillin and 100 μg/mL streptomycin, catalog number: 15140163, Thermo Fisher, USA) at 37 °C and 5% CO_2_. During the culture process, the medium was regularly changed, and the growth of cells was observed [31].

### 2.10. Synthesis and Characterization of G5-PBA

PBA (1821-12-1, Shanghai Aladdin Chemical Co., Ltd., Shanghai, China) and G5 PAMAM (Weihai Morning Source Molecular New Materials Co., Ltd., Weihai, Shandong, China) were synthesized in 70 °C DMSO (ST038, Shanghai BiyunTian Biotechnology Co., Ltd., Shanghai, China) using a molar ratio of 128:1. The mixture was then subjected to dialysis for 48 h, followed by additional extensive dialysis. Finally, the purified G5-PBA was freeze-dried.

### 2.11. Preparation and Characterization of siRNA@G5-PBA Complex

G5-PBA and 200 pmol siCOL1A2 (siB10825140144-1-5, purchased from Guangzhou Ruibo Biotechnology Co., Ltd.,Guangzhou China) were dissolved in water pretreated with diethyl pyrocarbonate. The mixture was prepared by adjusting the nitrogen-phosphorus ratio (N/P) and incubated at room temperature for 10 min to form the gene transfection complex. The particle size and zeta potential of the siRNA@G5-PBA complex were measured using a Zetasizer Nano-ZS (Malvern, UK). Subsequently, the samples were freeze-dried and loaded into a water gel on a copper grid for scanning electron microscope (SEM) analysis after freeze-drying the gel. The samples were characterized and observed using a scanning electron microscope (FEI QUANTA200, FEI Czech Republic sro, Brno-Černovice, Czech Republic) [32]. Gel electrophoresis with agarose gel was used to evaluate the degradation capability of siRNA@G5-PBA complexes after incubation with RNase (R598094, Shanghai Aladdin Chemical Co., Ltd., Shanghai, China).

### 2.12. Synthesis of a Multifunctional Hydrogel (OG/GCA)

Synthesis of OG: Dextran (4 g, S33186, Sourceleaf Biotech, Shanghai, China) was dissolved in deionized water at a concentration of 2% (*w*/*v*), and 4 g of NaIO4 (7790-28-5, Aladdin, Shanghai, China) was added to the solution. The reaction was conducted in a dark environment for 6 h. To terminate the oxidation reaction, 1 mL of ethylene glycol was added to the solution. The resulting mixture was dialyzed in deionized water for 3 days (MWCO 8–14 kDa) and subsequently freeze-dried to obtain oxidized dextran (ODEX). Then, 1 g of ODEX was dissolved in 100 mL of deionized water, and 1 g of Girard’s reagent T (123-46-6, Aladdin, Shanghai, China) was added. The solution was stirred at room temperature for 1 h, and the pH was adjusted to 5. After dialysis and freeze-drying, OG was obtained.

Synthesis of GCA: Gelatin (1 g, 9000-70-8, Aladdin, Shanghai, China) was dissolved in 100 mL of deionized water. Then, 364 mg of HCA (116-16-5, Aladdin, Shanghai, China), 382 mg of EDC (A638729, Aladdin, Shanghai, China), and 230 mg of NHS (1263093-76-0, Aladdin, Shanghai, China) were added. The pH was adjusted to 5.0, and the reaction was carried out overnight under nitrogen gas at room temperature. The resulting solution was dialyzed in deionized water for 3 days using a membrane purification tube (MWCO 8–14 kDa) and then freeze-dried to obtain gallic acid-coupled gelatin (GC). Subsequently, 1 g of GC was dissolved in 100 mL of deionized water, and 1.1 g of ADH (462605-73-8, Aladdin, Shanghai, China) was added. Then, 0.23 g of EDC was dissolved in 10 mL of deionized water, and 0.23 g of HOBt (123333-53-9, Aladdin, Shanghai, China) was added dropwise into the solution dissolved in 10 mL of DMSO. The pH was maintained at 5.0, and the reaction was carried out overnight. After 3 days of dialysis, GCA was obtained through freeze-drying.

The prepared OG polymers were dissolved in PBS at concentrations of 5%, 8.5%, and 12% (*w*/*v*), respectively. The synthesized GCA monomers were dissolved in PBS at concentrations of 10% and 15%. The OG and GCA polymers with different concentrations were mixed in equal volumes at room temperature. The resulting hydrogels were denoted as OG/GCA1 (5% OG and 10% GCA), OG/GCA2 (8.5% OG and 10% GCA), OG/GCA3 (8.5% OG and 15% GCA), and OG/GCA4 (12% OG and 15% GCA).

### 2.13. Gelation Time

The gelation time of the hydrogel was evaluated using the tilted vial method. Different concentrations of OG and GCA solutions were mixed in equal volumes in a vial, which was then inverted at 37 °C. The time required for the sol-gel transition, indicated by the cessation of flow, was recorded.

### 2.14. Rheological Testing

The rheological properties of the hydrogel were measured using an Anton Paar MCR302 rheometer, Anton Paar GmbH, Graz, Austria. Parallel plates with a diameter of 25 mm and a gap of 1 mm were used, and the temperature was set at 37 °C. The storage modulus (G′) and loss modulus (G″) were measured as rheological parameters. First, a frequency sweep test was conducted at a constant strain of 1% in the frequency range of 0.01 to 10 Hz. The average G′ at 1 Hz was calculated to evaluate the material’s rheological performance. Then, a shear rate sweep was performed at a fixed frequency of 1 Hz, with shear rates ranging from 0.1 to 1000 rad/s, to assess the shear thinning behavior of the hydrogel. To quantitatively evaluate the self-healing capability of the hydrogel, the gel was initially damaged by increasing the strain from 0.5% to 200% at 1 Hz, and then, while reducing the strain to 0.5%, a time scan test was conducted to monitor the self-healing behavior [32].

### 2.15. Swelling Testing

Frozen hydrogel samples weighing 400 μL (W0) were placed in 2 mL of PBS solution at a pH of 7.4 and a temperature of 37 °C. At predetermined time points, the surface of the swollen hydrogel was dried with filter paper, weighed, and labeled as Wt. The swelling percentage of the hydrogel was calculated using the formula: Swelling Percentage (%) = (Wt − W0)/W0 × 100%.

### 2.16. In Vitro Degradation Test

The degradation of hydrogel under physiological conditions was investigated. Freshly prepared 400 μL hydrogel samples were incubated in PBS (pH5.5) at 37 °C and 100 rpm. On days 7, 14, 21, and 28, the samples were weighed (Wt), and the degradation percentage of the hydrogel was calculated using the following formula: Residual weight (%) = Wt/W0 × 100%.

### 2.17. OC/GCA Adhesion Strength Testing

Shear testing was conducted to quantitatively measure the adhesive capacity of wet tissues using hydrogels. Different concentrations of OC/GCA hydrogel (400 μL) were applied between the surfaces of two pieces of porcine skin, with a bonding area of approximately 10 mm × 10 mm, followed by tissue maintenance at room temperature for 10 min. The adhesion performance was tested at a strain rate of 1 mm/min using a universal testing machine equipped with a 100 N load cell. The adhesion strength was calculated as the maximum stress divided by the bonding area.

### 2.18. Evaluation of Antimicrobial Activity of OC/GCA

*Staphylococcus aureus* (*S. aureus*), *Escherichia coli* (*E. coli*), and carbapenem-resistant Klebsiella pneumoniae (CRKP) were used for assessing the antimicrobial activity of OC/GCA hydrogels at different concentrations. Hydrogel samples (400 μL) were immersed in 1 mL PBS buffer containing 10^4^ CFU/mL bacteria and incubated at 37 °C with shaking at 200 rpm for 12 h. PBS with 10^4^ CFU/mL bacteria served as the control group. Subsequently, 10 μL of each sample was spread on Luria Bertani (LB) agar plates and incubated at 37 °C for 12 h. The colony counts on the plates were then recorded, and the antimicrobial rate was calculated using the formula: Antimicrobial rate (%) = (C0 − Ch)/C0 × 100%, where C0 and Ch represent the colony counts of the control and hydrogel groups, respectively. Additionally, bacterial culture media co-cultured with OG/GCA hydrogels were centrifuged at 12,000 rpm for 10 min to observe the morphology and status of bacteria by SEM to further evaluate the antimicrobial mechanism.

### 2.19. In Vitro Transfection Efficiency and Cytotoxicity Study of siRNA@G5-PBA

NP cells in each group were digested, resuspended, and adjusted to 1 × 10^5^ cells/mL. Then, 100 μL of the cell suspension was seeded in a 96-well plate and incubated overnight. For each group, 200 pmol of gel or siCOL1A2@G5-PBA@Gel complex was added and cultured for 1 day and 3 days. Cell viability was measured using the CCK-8 assay kit (C0041, Beyotime, Shanghai, China) according to the manufacturer’s instructions. During each measurement, 10 μL of CCK-8 detection solution was added, and the absorbance was measured at 450 nm using an enzyme immunoassay analyzer. Cell viability was then calculated [32].

For fluorescence microscopy, NP cells in each group were digested, resuspended, and adjusted to 1 × 10^5^ cells/mL. Then, 100 μL of the cell suspension was seeded in a 96-well plate and incubated overnight; each group was treated with 200 pmol of FAM-siCOL1A2, FAM-siCOL1A2-Lipo3000, or FAM-siCOL1A2@G5-PBA complex (FAM-siCOL1A2 customized by Dense Bio, Shanghai, China) was added to each group and cultured for 24 h. The fluorescence intensity was observed and measured under a fluorescence microscope (IX83-FV3000, Evident, Tokyo, Japan) with excitation and emission wavelengths of 492 nm and 518 nm, respectively.

### 2.20. Biocompatibility Assessment of siRNA@G5-PBA@Gel

First, 200 pmol of siRNA@G5-PBA complex was mixed with 200 µL of GCA for 10 min. Then, it was mixed with 200 µL of OG at room temperature to form the gene-loaded hydrogel (siRNA@G5-PBA@Gel). NP cells were seeded in a 24-well plate at a density of 5 × 10^4^ cells per well and incubated for 24 h. The gel or siRNA@G5-PBA@Gel was transferred to a cell culture insert (8 µm pore size) and co-cultured with NP cells for either 1 or 3 days. The cytotoxicity of the hydrogel was determined by measuring the absorbance at 450 nm using a microplate spectrophotometer (Bio-Tek, Thermo Fisher Scientific, USA).

To evaluate the direct effect of the hydrogel on cell attachment, 400 µL of gel or siRNA@G5-PBA@Gel was placed at the bottom of a 24-well plate, and each well was seeded with 1 × 10^5^ cells. On the third day, cells were stained with phalloidin (C2207S, Beyotime, Shanghai, China) to assess cell morphology and adhesion. Cell morphology and adhesion on the surface of the hydrogel were observed using a confocal microscope (Nikon A1 Ti, Nikon Instruments Inc., Tokyo, Japan).

### 2.21. Hemolytic Assay

The blood compatibility of the hydrogel was assessed using a hemolytic assay. Red blood cells were extracted from male Sprague-Dawley rats (8 weeks old) obtained from Beijing Weitonglihua Experimental Animal Technology Co., Ltd. (Beijing, China). The blood was centrifuged at 1000 rpm for 5 min, and the resulting red blood cell concentration was diluted to 2% (*v*/*v*) in PBS. Subsequently, 400 µL of gel or siRNA@G5-PBA@Gel was mixed with 1 mL of the red blood cell suspension and incubated at 37 °C with shaking at 100 rpm for 1 h. A positive control using 0.1% Triton X-100 and a negative control using physiological saline were included. The suspension was then collected and centrifuged at 1000 rpm for 10 min. The absorbance at 540 nm was measured using a microplate spectrophotometer (Bio-Tek, Thermo Fisher Scientific, USA). The hemolysis rate was calculated using the following equation: Hemolysis rate (%) = (As − Ab)/(At − Ab) × 100%, where As, Ab, and At represent the absorbance values of the supernatant for the hydrogel, physiological saline, and Triton X-100, respectively.

### 2.22. In Vitro pH-Responsive Release Experiment

First, cy5-labeled siRNA@G5-PBA nanocomplexes were prepared and loaded into OG/GCA hydrogel (Cy5-siRNA@G5-PBA@Gel). Next, the Cy5-siRNA@G5-PBA@Gel was incubated at 37 °C with shaking at 100 rpm in 2 mL of PBS at pH 7.4 or 5.5. The release of siRNA@G5-PBA was measured using Cytation3 (Bio-Tek, Winooski, VT, USA) with an excitation wavelength of 649 nm and an emission wavelength of 680 nm.

### 2.23. In Vivo Evaluation of siRNA@G5-PBA Release

400 µL of Cy5-siRNA@G5-PBA@Gel was subcutaneously injected into the dorsal region of three male C57BL/6J mice obtained from Beijing Weitonglihua Experimental Animal Technology Co., Ltd. (Beijing, China). The release levels of Cy5-siRNA@G5-PBA were detected at 0, 7, 14, 28, and 42 days post-injection using the Caliper IVIS Lumina II (CLS136341/F, Caliper Life Sciences, Hopkinton, MA, USA) with 649/680 nm detection wavelengths.

### 2.24. NP Cell Treatment

Gel or siCOL1A2@G5-PBA@Gel (400 μL) was deposited at the bottom of a 24-well plate. Next, 1 × 10^5^ rat NP cells were seeded onto the hydrogel surface and induced with 10 µg/mL of lipopolysaccharide (LPS, S1732, BiyunTian, Shanghai, China) to promote inflammation and degeneration of NP cells. The cells were then cultured continuously for 3 days.

### 2.25. Encapsulation of Rat NP Cells in siRNA@G5-PBA@Gel

5 × 10^5^ NP cells were suspended in 200 µL of OG solution containing siRNA@G5-PBA complexes. This mixture was then pre-mixed with 200 µL of GCA to form a uniformly mixed bioactive hydrogel suitable for gene-drug and cell loading. After encapsulation, the viability of the encapsulated NP cells in the culture medium was assessed using a live/dead staining assay, with a subsequent 7 or 14-day culture period. The live/dead cell staining was performed as follows: the live/dead assay kit (L10119, Invitrogen, Waltham, Massachusetts, USA) was used, following the manufacturer’s instructions. Calcein-AM and ethidium homodimer-1 were diluted to concentrations of 2 µM and 4 µM, respectively, in D-PBS. The NP cells in the culture medium were then removed, and the staining solution was added. The cells were incubated for 30 min in a cell culture incubator at 37 °C, 5% CO_2_, and appropriate humidity. The stained cells were imaged using an inverted fluorescence microscope (IMT-2, Olympus, Tokyo, Japan) to visualize live cells (green, Ex: 480 nm, Em: 530 nm) and dead cells (red, Ex: 530 nm, Em: 645 nm) [32].

### 2.26. Construction and Treatment of a Rat Model of Lumbar Intervertebral Disc Prolapse

The animal experiments were conducted following the approved protocol of our Animal Experimentation Committee. A total of 48 eight-week-old male Sprague-Dawley rats weighing between 270 and 300 g (Beijing Vita Biological Technology Co., Ltd., Beijing, China) were used for in vivo experiments.

Establishment of the IVDD model: Following a 12-h fasting period and a 4-h water deprivation, the rats were intraperitoneally injected with 0.3% pentobarbital anesthesia. Subsequently, a 20-gauge needle was used to puncture the intervertebral discs (puncture points: Co6/7, Co7/8, Co8/9). The needle traversed the entire intervertebral disc and was rotated 180 degrees, held for 30 s, then rotated for 5 s, and held for another 30 s to stabilize it. The control group underwent the same surgical procedure, exposing the peritoneum of the lumbar spine but without needle puncture.

The experiment included six groups, each consisting of eight rats: Sham group (exposed to lumbar spinal dura mater surgery without needle puncture), IVDD group, Gel group (treated with gel after IVDD modeling), Treatment group (received acupuncture treatment after IVDD modeling), siCOL1A2@G5-PBA@Gel group (treated with siCOL1A2@G5-PBA@Gel after IVDD modeling), and Treatment + siCOL1A2@G5-PBA@Gel group (received acupuncture treatment and siCOL1A2@G5-PBA@Gel combined treatment after IVDD modeling). In the first week following the establishment of the puncture model, intradiscal injection and acupuncture treatment were performed. Gel injection involved injecting 20 µL of different gels into each disc using a 21G needle. After successful modeling, rats in the acupuncture group received acupuncture intervention at acupoints based on the “Commonly Used Acupoint Names and Locations for Experimental Animals Part 2: Rats” published by the Chinese Acupuncture Association. The acupoints included Shenshu, Dachangshu, Huan Tiao, Wei Zhong, Yanglingquan, and Ashi Point. The rats were placed in a prone position, limbs fixed, selected acupoints exposed, disinfected, and needling performed using specific needle sizes and angles according to the guidelines, with manipulation techniques applied. Acupuncture was administered once daily for 30 min per session, for 7 sessions constituting one course, with a total of 4 courses. In the eighth week post-modeling, pain tests and imaging examinations were conducted before collecting tissue and peripheral blood samples for subsequent experiments [33].

### 2.27. Pain Behavior Testing

Four pain-related behavior tests were conducted using a force gauge (Bioseb, Pinellas Park, FL, USA) to measure the pain threshold and evaluate pressure-induced hyperalgesia. Rat subjects were subjected to pressure by applying the sensor tip to the skin above L4/5 at a rate of 50 g per second until a vocalization response was observed. The spontaneous activity level of rats, including walking distance, total activity time, and maximum velocity, was evaluated using a wheel activity device (Bioseb, USA), recording rotations in two directions [34].

### 2.28. Radiological Examination

X-ray examination: Pre- and post-operatively, intervertebral disc X-ray and magnetic resonance imaging (MRI) scans were performed on rats. Prior to the examination, rats were anesthetized using 2% isoflurane (1 L/min), and imaging was conducted while the rats were fixed in the prone position. X-ray images (Philips, exposure time: 40 s, distance: 40 cm, 3 mA/35 kV) were used to measure the height of the intervertebral disc before and after surgery, represented by the intervertebral disc height index (DHI). The measurements were carried out using Surgimap 2.3.2.1 (Nemaris Inc., New York, NY, USA) software.

MRI scanning: Magnetic resonance imaging sagittal plane scans of the rat intervertebral disc were conducted using a superconductive MRI system (Siemens TRIO, TR/TE = 2556/85 ms, FOV 22.0 cm, NSA2, SCAN 130 s, Thick 1.0 mm). The T2 signal intensity values of the experimental and control groups’ intervertebral disc NP were measured using the Siemens system’s built-in tools. The measurement area was 0.7 mm^2^, repeated three times, and the average value was taken. The MRI images were graded according to the modified Thomson classification (I–IV) (I: Normal, II: Minimal signal intensity decrease with apparent narrowing of high signal areas, III: Moderate signal intensity decrease, IV: Severe signal intensity decrease).

### 2.29. Histological Staining of Tissues

Hematoxylin and Eosin Staining: Samples were stained using the Hematoxylin and Eosin staining kit (C0105S, Beyotime, Shanghai, China). Initially, samples were dehydrated in a gradient series of ethanol (70%, 80%, 95%, and 100% ethanol solutions for 1 h each) and then cleared in xylene overnight. Subsequently, the samples were infiltrated with paraffin overnight, transferred to the paraffin embedding station, and 5 μm thick consecutive sections were prepared using a microtome. The sections were then baked, deparaffinized, washed in water, and stained with Hematoxylin. After washing the samples in distilled water, they were immersed in 95% ethanol and counterstained with Eosin. Finally, the sections underwent differentiation in 70% hydrochloric acid ethanol, dehydration, and clearing in xylene before being mounted with a neutral mounting medium. Morphological changes in the rat intervertebral disc tissues were observed under an optical microscope. Selected sections were required to include the three-layered structure of cartilaginous endplates, annulus fibrosus, and nucleus pulposus in the transverse sections of the disc tissue [35].

Safranine O-fast green Staining: The samples were stained using a staining kit (G1371, Beijing Solabao Technology Co., Ltd., Beijing, China) with safranine O-fast green to observe the distribution of proteoglycans in the intervertebral disc. According to the instructions, the specific procedure was as follows: firstly, the samples were stained with safranine O for 1 h, and the excess dye was washed away with distilled water. Then, the sections were subjected to a gradient of 50% to 80% ethanol for decolorization. Subsequently, the sections were stained in solid green dye for 60 s and dehydrated in absolute ethanol. Finally, the sections were cleared in xylene for 5 min and immediately mounted with neutral resin [31].

Based on H&E and safranin O-fast green staining, intervertebral discs were scored for degenerative changes across five categories, ranging from 0 (no degeneration) to 15 (severe degeneration). For NP morphology, scores ranged from 0 (round NP, >75% disc area) to 3 (round NP, <25% disc area). NP cellular morphology was scored from 0 (evenly distributed star-shaped cells) to 3 (large round cells separated by dense proteoglycan). AF morphology was scored from 0 (intact collagen) to 3 (inward bulging/rupture/snake-like fibers occupying > 50% of the disc surface). AF cellular morphology was scored from 0 (fibroblasts > 90% of cells) to 3 (chondrocytes > 75% of cells). The boundary between NP and AF was scored from 0 (normal) to 3 (severe interference) [36].

### 2.30. Immunohistochemical Staining

Paraffin was cooled on ice or in a 4 °C refrigerator, and tissue sections were embedded. After allowing the paraffin-embedded sections to sit overnight, the slides were placed in a 60 °C oven for 20 min. Sections were then soaked in xylene twice for 10 min each. Rehydration was performed by soaking in absolute alcohol for 5 min, followed by another 5-min soaking in fresh absolute alcohol. The sections were further hydrated sequentially in 95% and 70% ethanol for 10 min each and finally rinsed with distilled water for 5 min. The sections were immersed in citrate buffer (pH 6.0) and heated in a microwave on high power for 8 min, then cooled to room temperature. Slides were washed three times with PBS (pH 7.2~7.6) for 3 min each. To inactivate endogenous enzymatic activity, 3% H_2_O_2_ was added and incubated at room temperature for 10 min. After washing three times with PBS, the sections were blocked with normal goat serum (E510009, Biotech Biotechnology Co., Ltd., Shanghai, China) for 20 min at room temperature. The sections were then incubated overnight at 4 °C with rabbit anti-COL1A2 primary antibody (PA5-50938, 1:1000, Thermo Fisher, UK), followed by washing with PBS. Next, the sections were incubated with goat anti-rabbit IgG secondary antibody (ab6721, 1:5000, Abcam, Cambridge, UK) for 30 min. SABC (P0603, Beyotime, Shanghai, China) was added and incubated at 37 °C for 30 min. DAB staining (P0203, Beyotime, Shanghai, China) was performed for 6 min, and the sections were counterstained with hematoxylin for 30 s. Dehydration was performed using 70%, 80%, 90%, 95% ethanol, and absolute ethanol for 2 min each, followed by two 5-min xylene washes. The tissues were sealed with neutral resin and observed under a brightfield microscope (BX63, Olympus, Japan). For each slide, five random high-power fields were selected for observation. The Image-Pro Plus 6.0 software was used to count the number of positive cells and total cells in each of the five fields. Subsequently, the average number of positive cells and total cells was calculated for the five fields, and the ratio of the average number of positive cells to the average number of total cells was determined [37].

### 2.31. TUNEL Assay for Cell Apoptosis Detection

Tissues were stained using the TUNEL staining kit (C1086, Beyotime, Shanghai, China). In brief, the intervertebral disc tissues were fixed in 4% paraformaldehyde for 30 min and then washed three times with PBS. The tissues were permeabilized with PBS containing 0.3% Triton X-100 for 3 min at room temperature, followed by incubation for 5 min. After two PBS washes, 50 μL of TUNEL detection reagent was added, and the tissues were incubated under light-protected conditions at 37 °C for 60 min. Then, three PBS washes were performed, followed by a 10-min re-staining with DAPI (10 μg/mL, C1002, Beyotime, Shanghai, China). Finally, the samples were sealed with an anti-fluorescence quenching mounting medium and observed under a fluorescence microscope. The apoptosis ratio of each group was quantified using Image-Pro Plus 6.0 software [38].

### 2.32. Western Blot

Tissues and cells were lysed using RIPA lysis buffer (P0013B, Beyotime, Shanghai, China) with 1% PMSF. Total protein was extracted according to the manufacturer’s instructions, and the concentration was determined using a BCA assay kit (P0011, Beyotime, Shanghai, China). Protein concentrations were adjusted to 1 μg/μL, with each sample containing 100 μL. Samples were boiled at 100 °C for 10 min to denature the proteins and then stored at −80 °C. SDS-PAGE gels (8–12%) were prepared according to the target protein size, and 50 μg of protein was loaded per well. Electrophoresis was performed at 80 V for 2 h, followed by transfer to a PVDF membrane (1620177, Bio-Rad, Hercules, California, USA). The membrane was blocked with 5% skimmed milk in 1xTBST for 1 h at room temperature and then washed three times with 1xTBST. Primary antibodies—COL1A2 (PA5-50938, 1:1000, Thermo Fisher, UK), MMP3 (ab52915, 1:1000, Abcam, UK), MMP13 (ab39012, 1:1000, Abcam, UK), IL-1β (ab315084, 1:1000, Abcam, UK), TNF-α (ab307164, 1:1000, Abcam, UK), and β-actin (ab7817, 1:1000, Abcam, UK)—were incubated overnight at 4 °C. The membrane was then washed with 1xTBST and incubated with HRP-conjugated goat anti-rabbit IgG (ab6721, 1:5000, Abcam, UK) or goat anti-mouse IgG (ab205719, 1:5000, Abcam, UK) at room temperature for 1 h. After washing, the membrane was incubated in ECL solution (1705062, Bio-Rad, USA) for 1 min and exposed using the Image Quant LAS 4000C gel imaging system (GE HealthCare, Chicago, IL, USA). β-actin served as the internal control, and the relative expression levels were calculated as the ratio of the gray values between the target and reference bands. Protein expression levels were measured for each group in triplicate [39].

### 2.33. Expression of Inflammatory Factors Detected by ELISA

Serum samples from each group of rats were collected and analyzed for TNF-α (ab208348, Abcam, USA) and IL-1β (ab197742, Abcam, USA) expression using ELISA assay kits. Briefly, standards and samples were added to wells and incubated, allowing protein binding to immobilized antibodies. Subsequently, washing was carried out, followed by the addition of biotinylated specific antibodies. After a specific duration, unbound biotinylated antibodies were washed away, and HRP conjugate was added. Further washing was performed before adding 3,3′,5,5′-Tetramethylbenzidine (TMB) substrate. The addition of TMB resulted in a blue color, which then turned yellow upon the addition of the stop solution. The optical density (OD) was measured at 450 nm. The expression of each inflammatory factor was determined by calculating protein concentration using the standard curve [34].

### 2.34. Statistical Analysis

Descriptive statistics were initially performed to summarize the data, including the calculation of mean and standard deviation (Mean ± SD), providing an overview of the basic characteristics of each group’s data. The Shapiro-Wilk test was used to assess the normality of the dataset for normality testing purposes. Levene’s Test was employed to assess the equality of variances among experimental groups, which determined the appropriate statistical methods for subsequent analysis. When the data were normally distributed and had equal variances, independent-sample *t*-tests were used for comparisons between two groups, while one-way analysis of variance (ANOVA) was applied for comparisons among multiple groups. Nonparametric tests, such as the Mann-Whitney U test for two-group comparisons or the Kruskal-Wallis test for multiple-group comparisons, were utilized when the data did not meet normal distribution or equal variance assumptions. In the case of significant differences observed after ANOVA, Dunnett’s T3 or Tukey’s HSD post-hoc tests were conducted for multiple comparisons to determine specific intergroup differences. The significance level for all statistical tests was set at *p* < 0.05. Charts and graphs (such as bar graphs, box plots, etc.) were used to visually present the results of the statistical analyses.

## 3. Results

### 3.1. Acupuncture Promotes Restoration and Regeneration of IVDD in Rats

**Figure 1:**
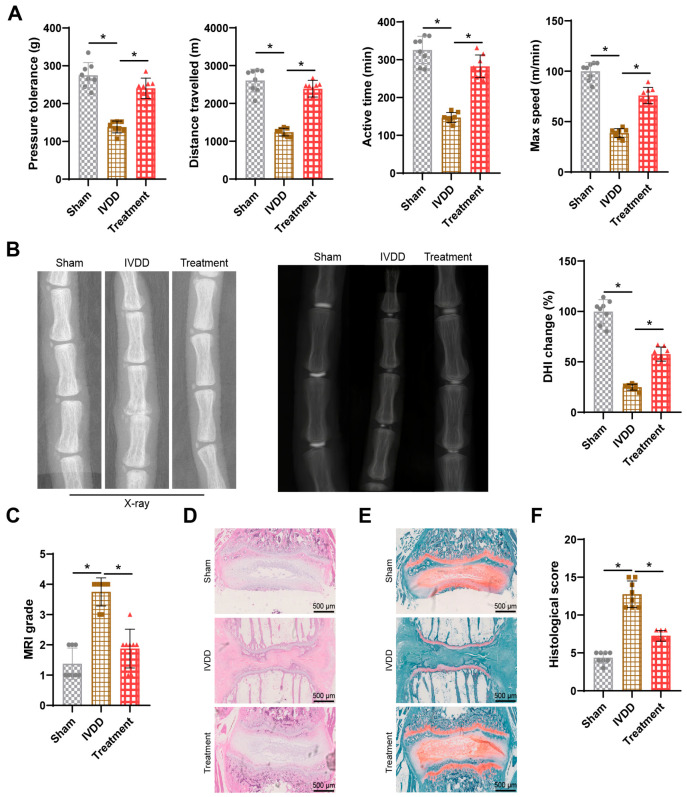
**Effects of acupuncture treatment on IVD rats.** Note: (**A**) Results of pain response behavior testing in rats; (**B**) Representative X-ray images of rat intervertebral discs and statistical values of DHI; (**C**) MRI images of rat intervertebral discs and quantitative scoring; (**D**) H&E staining of rat intervertebral discs in different treatment groups (scale bar = 500 μm); (**E**) Safranine O-fast green staining of rat intervertebral discs in different treatment groups, where green represents bone tissue and red represents cartilage tissue (scale bar = 500 μm); (**F**) Histological scores of intervertebral discs in each group. N = 8, * indicates *p* < 0.05 compared between the two groups.

Previous research has demonstrated the efficacy of acupuncture in treating IVDD. Following acupuncture treatment, local oxidative-reductive potential in dogs with IVDD was improved, leading to increased energy metabolism and providing some relief from pain [40]. To confirm the impact of acupuncture on IVDD, we measured pain responses in rats, including pressure sensitivity, walking distance, total activity time, and maximum speed. The results revealed that compared to the Sham group, IVDD rats exhibited significant decreases in pressure sensitivity, walking distance, total activity time, and maximum speed, while the acupuncture treatment group showed significant improvements in these parameters compared to the IVDD rats (Figure 1A).

Imaging evaluation and histological sections are reliable indicators reflecting IVDD or regeneration [41]. After 7 weeks of treatment, we used an X-ray examination to observe changes in intervertebral disc height in rats (Figure 1B). The intervertebral disc height of IVDD rats was significantly reduced compared to the Sham group; however, the acupuncture treatment group showed significant restoration of intervertebral disc height in IVDD rats compared to the IVDD group. MRI also demonstrated that acupuncture treatment promoted the regeneration of degenerated intervertebral discs in rats, delaying the degeneration process and restoring the water content of the intervertebral discs to normal levels (Figure 1C).

Histological staining of rat intervertebral disc tissues with H&E and safranine O-fast green staining revealed that compared to the Sham group, the IVDD group displayed ruptured and disordered NP, fuzzy boundaries between the NP and annulus fibrosus, and a significant decrease in cartilaginous components within the tissue. In contrast, compared to the IVDD group, the acupuncture treatment group showed improvement in structural damage and degeneration of intervertebral disc tissues in IVDD rats, with clear boundaries between the NP and annulus fibrosus and reduced loss of cartilaginous components (Figure 1D–F). These findings collectively demonstrate that acupuncture promotes the repair and regeneration of IVDD in rats.

### 3.2. Acupuncture Inhibits Inflammation and Cell Apoptosis in the NP

**Figure 2:**
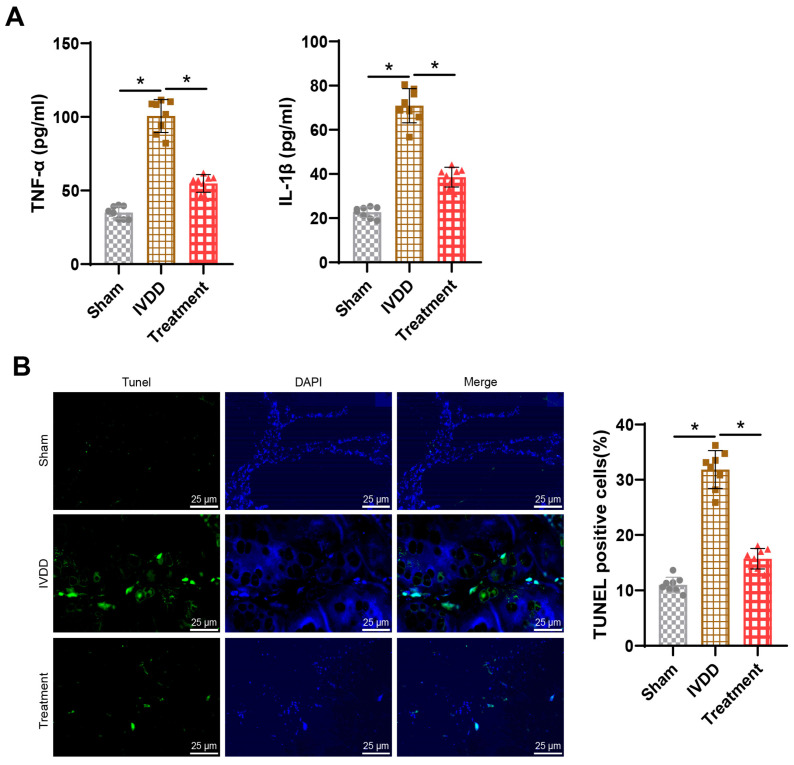
**Combined effects of siCOL1A2@G5-PBA@Gel and acupuncture treatment on IVD rats.** Note: (**A**) ELISA assessment of TNF-α and IL-1β inflammatory factor expression in rat serum; (**B**) TUNEL staining to analyze cell apoptosis in rat NP tissue (scale bar = 25 μm), * *p* < 0.05.

Research has indicated the crucial role of inflammation in IVDD. Enhanced inflammatory responses can lead to impaired cellular function within the intervertebral disc, affecting cellular metabolism and biomechanical properties thereby hastening the degenerative process [42]. ELISA analysis revealed that compared to the Sham group, expression levels of the inflammatory factors TNF-α and IL-1β were significantly elevated in the serum of rats in the IVDD group; however, following acupuncture treatment, there was a reduction in the expression of these inflammatory factors in the serum, indicating a decrease in the inflammatory response (Figure 2A). Furthermore, TUNEL assay results demonstrated a significant increase in cell apoptosis in the NP of rats in the IVDD group compared to the Sham group; notably, after acupuncture treatment, a marked decrease in cell apoptosis within the NP was observed (Figure 2B).

These findings collectively suggest that acupuncture is effective in inhibiting inflammation and cell apoptosis in the NP.

### 3.3. Key Gene Selection and Evaluation: COL1A2 Holds Important Diagnostic Value in IVDD

Previous studies have shown that acupuncture has a certain therapeutic effect on IVDD. However, it often only provides temporary relief of pain and symptom alleviation without curing the condition. Therefore, this study aimed to explore a combinational treatment approach targeting acupuncture therapy. Existing research indicates that gene therapy based on small interfering RNA (siRNA) is an emerging treatment method targeting specific pathological pathways or related diseases [43]. The accumulation of inflammatory mediators not only damages intervertebral disc cells but may also stimulate the surrounding nerve roots, leading to pain and functional impairments. Thus, managing and regulating the inflammatory response may be a key therapeutic strategy in slowing the progression of IVDD [44]. Therefore, to identify therapeutic targets, we initially screened for inflammation-regulating genes involved in IVDD through bioinformatics analysis.

Initially, differential gene analysis was conducted on the IVDD dataset GSE15227, yielding 1559 significant DEGs using the Limma package, including 1112 upregulated DEGs and 447 downregulated DEGs (Figure S1A). A heatmap displayed the gene expression patterns in IVDD and control groups (Figure S1B). By searching the GeneCards database for genes related to IVDD and inflammation with a Relevance score > 1 as the screening criterion, we identified 886 IVDD-related genes and 4305 inflammation-related genes. Venn analysis was performed on the DEGs from GSE15227 and the genes retrieved from the GeneCards database, resulting in the selection of 60 target genes involved in inflammation regulation in IVDD (Figure S1C).

Further analysis of the selected 60 target genes was carried out through GO functional analysis and KEGG pathway analysis. The GO functional analysis revealed that the candidates were involved in BP, such as vascular development, wound healing, and ossification; in CC, including ECM structures, collagen trimers, and basement membranes; and in MF, such as structural molecule activity, growth factor binding, and glycosaminoglycan binding (Figure S1D–F). KEGG pathway analysis indicated that the 60 candidate target genes were associated with protein digestion and absorption, the PI3K-Akt signaling pathway, and the HIF-1 signaling pathway (Figure S1G).

Using the PPI network analysis method, the relationships among these 60 key genes were studied. Subsequently, through STRING database submission of the target proteins and visualization using Cytoscape software, a complex PPI network illustrating the interactions among the targets was constructed (Figure S2A). The Degree method in Cytohubba identified the top 10 genes based on their Degree ranking, where COL1A1, HIF1A, CTNNB1, DCN, FN1, SPARC, COL1A2, IL6, COL3A1, and BGN had the highest Degree values (Figure S2B).

To further narrow down the selection to the most meaningful key genes, a combination of two machine learning methods (LASSO and RF) was employed. Initially, the LASSO regression method identified 5 potential candidate genes (IL6, COL1A1, COL1A2, SPARC, and COL3A1) (Figure S3A,B). Subsequently, the RF algorithm was used to rank these 10 target genes, and the results, as shown in Figure S3C, led to the selection of the top 5 genes for further analysis (COL1A1, COL1A2, SPARC, HIF1A, and DCN) (Figure S3D). A Venn diagram was plotted to display the overlap between the 5 genes identified through LASSO and the top 5 genes from the RF ranking. Ultimately, 3 key genes were selected, namely SPARC, COL1A1, and COL1A2 (Figure S3E).

To further evaluate the diagnostic efficacy of these 3 key genes in IVDD, ROC curves were plotted, and AUC values were calculated using the GSE15227 dataset. Among them, COL1A2 exhibited an AUC value of 1.000, indicating its high diagnostic accuracy, implying its potential value in disease state recognition (Figure S3F). Comparative analysis of COL1A2 expression levels in the IVDD group and the control group from the GSE15227 dataset revealed a significant increase in COL1A2 expression in the degeneration group in the training set (Figure S3G). COL1A2 (Collagen, Type I, Alpha 2) is a crucial component of type I collagen and is known to be more rigid and stiff compared to type II collagen. This alteration may lead to reduced elasticity of the intervertebral disc, weakening its buffering and weight-bearing capacities and accelerating the degeneration process [45]. Additionally, it regulates the composition between cells and the ECM, influencing cellular migration and tissue remodeling processes during inflammation [46]. In the process of IVDD, there are significant changes in the composition of the ECM, especially an increase in type I and type III collagen content, while type II collagen decreases. The increase in type I collagen content may be associated with inflammation-related responses, heightened pain perception, and symptoms such as nerve root compression [47].

**Figure 3:**
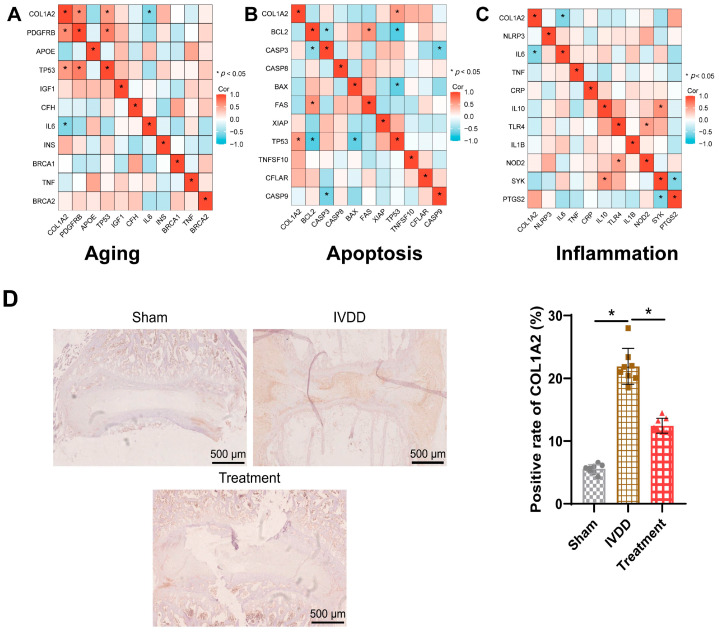
**COL1A2 is a key target for IVDD therapy.** Note: (**A**) Pearson correlation analysis of COL1A2 was conducted for the top 10 genes closely related to aging; (**B**) Pearson correlation analysis of COL1A2 was performed for the top 10 genes closely related to apoptosis; (**C**) Pearson correlation analysis of COL1A2 was carried out for the top 10 genes closely related to inflammation; (**D**) IHC detection of COL1A2 protein expression in rat NP tissues of each group (scale bar = 500 μm). N = 8, * indicates a significant difference between the two groups, with *p* < 0.05.

To further explore the potential mechanisms of COL1A2 in IVDD, we conducted a Pearson correlation analysis between COL1A2 expression levels and the top 10 genes related to apoptosis, aging, and inflammation. The results revealed significant positive correlations between COL1A2 and aging-related genes PDGFRB and TP53, with a significant negative correlation with IL6 (Figure 3A). Additionally, a significant positive correlation was observed between COL1A2 and the apoptosis-related gene TP53 (Figure 3B), while a significant negative correlation was found with the inflammation-related gene IL6, indicating a potential link between COL1A2 and inflammation (Figure 3C). Furthermore, immunohistochemical analysis of COL1A2 protein expression in the NP tissue of IVDD model mice demonstrated a significant increase in COL1A2 protein expression in the IVDD group compared to the Sham group; however, after acupuncture treatment, there was a significant decrease in COL1A2 protein expression in the NP tissue of the IVDD group (Figure 3D).

In conclusion, COL1A2 plays a significant regulatory role in the response to inflammation, as well as in the processes of aging and apoptosis, within degenerated intervertebral disc tissues.

### 3.4. siCOL1A2@G5-PBA@Gel Demonstrates Excellent Biocompatibility and Low Hemolysis Rate

Hydrogels, including cryogel and hydrogel, are emerging therapeutic materials known for their exceptional biocompatibility and sustained release properties, thus extensively researched and applied in the field of treatment [11,12]. Within the precursor chains of hydrogels, Oligosaccharide Glue (OG) stands out as a natural polysaccharide with excellent biocompatibility and biodegradability [48]. Serving as an ECM derivative, Gelatin Containing Arginine-Glycine-Aspartic Acid (GCA) is rich in RGD sequences, facilitating cell adhesion, proliferation, and remodeling [49]. The OG/GCA hydrogels exhibit rapid gelation, injectability, and self-healing properties, along with robust adhesion hemostatic and antibacterial abilities [50]. Moreover, as previously demonstrated [50], the G5/GCA gel loaded with siP65 can alleviate the inflammatory storm and significantly enhance the regeneration of IVD when used in combination with cell therapy. Hence, through a straightforward chemical reaction, we modified PBA to G5 PAMAM and synthesized the gene carrier G5-PBA dendrimer. Subsequently, we loaded siRNA COL1A2 (siCOL1A2) onto G5-PBA for transfection.

We prepared siRNA@G5-PBA complexes using various N/P ratios. G5-PBA, possessing positively charged amino groups, can self-assemble with the negatively charged phosphate groups on siRNA to form complexes (Figure S4A). Transmission electron microscopy (TEM) revealed the typical nanoscale structure of siRNA@G5-PBA complexes (Figure S4B) with a diameter of approximately 100 nm (Figure S4C). Furthermore, compared to naked siRNA, G5-PBA demonstrated stability against RNase degradation, indicating the RNase-resistant property of siRNA@G5-PBA (Figure S4D). To further assess transfection efficiency, we employed the commercial transfection reagent Lipo 3000 (Thermo Fisher Scientific, headquartered in Waltham, MA, USA) as a positive control and assessed the fluorescence intensity of FAM-labeled siRNA in NP cells after various treatments. Results exhibited higher FAM fluorescence in cells transfected with siCOL1A2@G5-PBA compared to those transfected with G5-PBA using Lipo 3000 (Figure S4E). Western blot analysis (Figure S4F) demonstrated a significant reduction in COL1A2 protein expression in cells transfected with siCOL1A2@Lipo 3000 and siCOL1A2@G5-PBA compared to siCOL1A2 alone. Importantly, the decrease in COL1A2 protein expression was more pronounced in cells transfected with siCOL1A2@G5-PBA than in those transfected with siCOL1A2@Lipo 3000, indicating the superior and stable ability of siCOL1A2@G5-PBA in reducing COL1A2 protein expression in NP cells.

Using OG and GCA as precursor polymers, a hydrogel was formed through dynamic cross-linking at room temperature by mixing equal volumes, incorporating acyl hydrazone bonds, imine bonds, and hydrogen bonds (Figure S5A). As depicted in Figure S5B, the hydrogel demonstrated resilience to both compression and stretching. Subsequently, hydrogel samples of varying concentrations were prepared, and gelation time was assessed using a tilting test with small vials. The results indicated that gelation occurred within 20 s across all groups, with the gelation rate accelerating with increasing concentrations of OG or GCA (Figure S5C). The mechanical properties of the hydrogel are crucial for its application as a biomaterial. By evaluating the mechanical properties using rheology and compression testing, frequency sweeps revealed that the storage modulus (G′) remained relatively constant throughout the process, while the loss modulus (G″) increased between 0.01 Hz and 10 Hz with escalating polymer concentrations, indicating the stability of the viscoelastic hydrogel network under the typical loading frequency for IVD (around 4–5 Hz) (Figure S5D). Moreover, the average G′ at 1 Hz rose with increasing polymer concentrations (Figure S5E). Compressive analysis findings were consistent with rheological results, indicating that the enhancement in mechanical performance was attributed to an increase in cross-linking density (Figure S5F). The swelling behavior of the hydrogel, as illustrated in Figure S5G, revealed that as the cross-linking density increased, the swelling ratio of the hydrogels decreased. When immersed in PBS and subjected to agitation at a rate of 100 rotations per minute at 37 °C, the hydrogel degraded over time due to the rupture of the dynamic bonds. Additionally, the hydrogel demonstrated stability for over 4 weeks (Figure S5H), showcasing an ideal characteristic for long-term gene-drug delivery and in vivo applications. The use of scanning electron microscopy (SEM) revealed a representative porous structure of hydrogels, where an increase in crosslinking density led to a decrease in pore size (Figure S5I). Moreover, shear adhesion tests were conducted on fresh pig skin to quantify the wet tissue adhesive strength of the hydrogels. The results indicated adhesive strengths of 2.8, 4.4, 5.7, and 4.1 kPa for OG/GCA1, OG/GCA2, OG/GCA3, and OG/GCA4 hydrogels, respectively, with OG/GCA3 exhibiting the highest adhesive strength (Figure S5J,K). Infection caused by implanted materials can impair tissue healing. While antibiotics can be used to combat bacterial infections, the risk of developing resistance is high [51]. Cationization of the polymers with Girard’s T reagent enables electrostatic interactions with the negatively charged bacterial cell membranes, effectively inactivating pathogens and imparting antimicrobial properties to the hydrogels. Therefore, further assessment was carried out on the antibacterial activity of hydrogels of different concentrations, revealing that all OG/GCA hydrogel groups inhibited the growth of *Staphylococcus aureus*, *Escherichia coli*, and carbapenem-resistant Klebsiella pneumoniae (CRKP) on agar plates. OG/GCA2, 3, and 4 hydrogels exhibited antibacterial rates exceeding 99% against *Staphylococcus aureus*, *Escherichia coli*, and CRKP bacteria (Figure S5L). Considering the physical properties, adhesive strength, and antibacterial activity, we ultimately chose OG/GCA3 formulation (comprising 8.5% OG and 15% GCA) for further experimentation.

**Figure 4:**
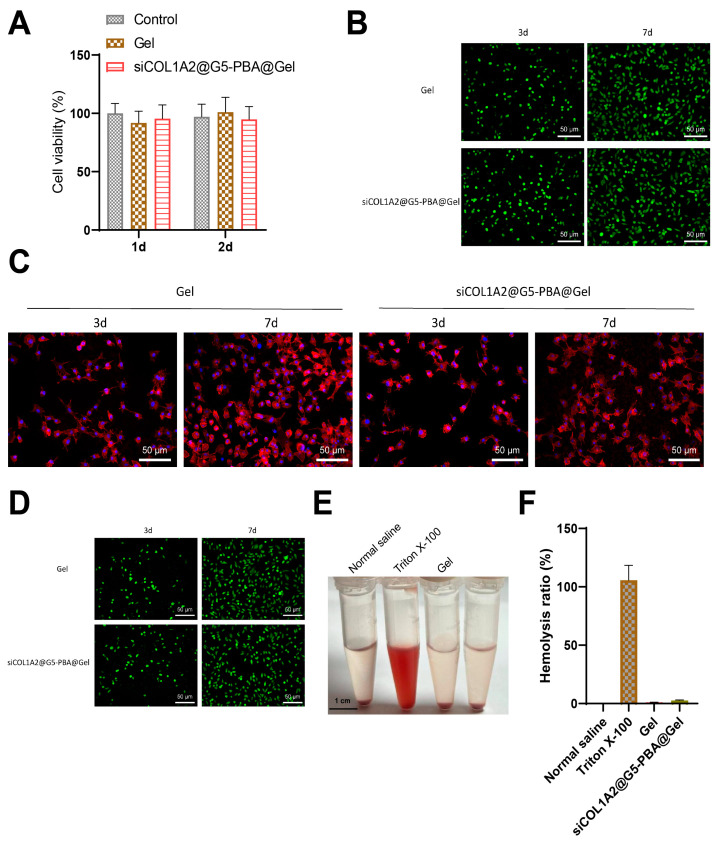
**Biocompatibility of siCOL1A2@G5-PBA@Gel.** Note: (**A**) Viability of rat NP cells co-cultured with the complex measured by CCK-8 assay; (**B**) Live/dead staining of NP cells cultured on hydrogel surface for 3 and 7 days (scale bar = 50 μm), green fluorescence represents live cells, red fluorescence represents dead cells; (**C**) Phalloidin staining of NP cells cultured on hydrogel surface for 3 and 7 days (scale bar = 50 µm), red fluorescence represents Phalloidin; (**D**) Live/dead staining of NP cells encapsulated in gel or siCOL1A2@G5-PBA@Gel, after 7 and 14 days (scale bar = 50 µm), green fluorescence represents live cells, red fluorescence represents dead cells; (**E**,**F**) Results of blood compatibility and hemolysis assay for gene-loaded multifunctional hydrogel, experiments were replicated 3 times.

Subsequently, we synthesized a multifunctional hydrogel with targeted siRNA delivery, biocompatibility, and antibacterial activity. This hydrogel, named siCOL1A2@G5-PBA@Gel, was formed by blending the gene-drug siCOL1A2@G5-PBA with the precursor polymer to enable gene-drug loading. Biocompatibility studies were conducted, and CCK-8 assay results revealed no significant alteration in cell viability of NP cells after co-culture for 1 and 3 days with gel or siCOL1A2@G5-PBA@Gel (Figure 4A). Live/dead staining revealed that NP cells maintained high viability on the surface of gel or siCOL1A2@G5-PBA@Gel (Figure 4B). Additionally, filamentous actin staining was employed to monitor the growth of NP cells on the hydrogel surface; as shown in Figure 4C, cells displayed spindle morphology after 3 and 7 days of culture. Figure 4D demonstrated that NP cells encapsulated in siCOL1A2@G5-PBA@Gel exhibited a certain proliferative activity after 3 and 7 days, with no apparent cell death.

The blood compatibility of the hydrogel was assessed through a hemolysis test, using Triton X-100 as the positive control and physiological saline as the negative control. As depicted in Figure 4E,F, both gel and siCOL1A2@G5-PBA@Gel exhibited similar coloration to the physiological saline group, with a lower hemolysis rate observed in the siCOL1A2@G5-PBA@Gel group.

The results indicate that the drug-loaded hydrogel system is non-toxic, displays good cell compatibility, and has a low hemolysis rate, meeting the fundamental requirements for biomedical internal applications.

### 3.5. Cy5-siCOL1A2@G5-PBA@Gel Exhibits Persistent Gene-Drug Release Capability and Suppresses Inflammatory Response

**Figure 5:**
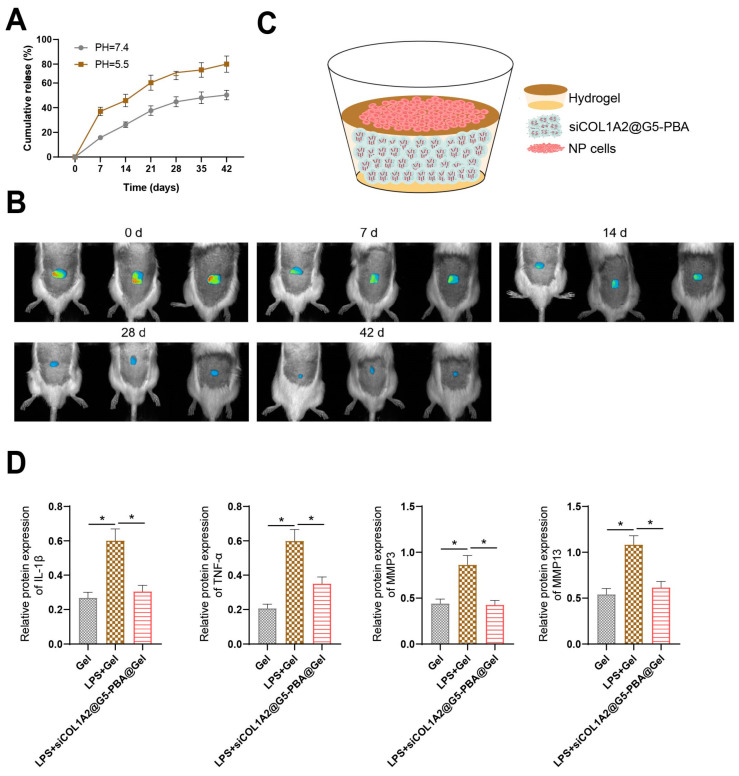
**In vitro and in vivo gene-drug release and effects on NP cell inflammation of multifunctional hydrogel.** Note: (**A**) Cumulative release curves of Cy5-siCOL1A2@G5-PBA@Gel hydrogel measured by PBS at pH 5.5 and 7.4 conditions (n = 3); (**B**) Real-time fluorescence imaging of Cy5-siCOL1A2@G5-PBA@Gel gel after subcutaneous injection in rats, observing its release time; (**C**) Illustration showing the seeding of NP cells on the surface of Cy5-siCOL1A2@G5-PBA@Gel; (**D**) Protein levels of IL-1β, TNF-α, MMP3, and MMP13 in NP cells from different groups detected by WB. *, indicate significant differences between groups (*p* < 0.05). Cell experiments were replicated three times.

Cy5-labeled siRNA was used to evaluate the release profile of siCOL1A2@G5-PBA@Gel in vitro and in vivo. In the in vitro experiments, Cy5-siCOL1A2@G5-PBA@Gel demonstrated sustained release of the gene-drug for over 28 days when incubated in PBS with shaking at 100 pm, at both pH 5.5 and 7.4. Notably, the release of siCOL1A2 was higher and faster in Cy5-siCOL1A2@G5-PBA@Gel at pH 5.5 (Figure 5A). Intravital disc degeneration (IVDD) is characterized by an acidic pathologic microenvironment [52,53]. The acidic reactivity of this hydrogel holds significant potential for achieving efficient and precise drug release. Real-time fluorescence imaging demonstrated that following subcutaneous injection on the back, Cy5-siCOL1A2@G5-PBA@Gel exhibited sustained local release in vivo for over 28 days (Figure 5B). These findings indicate the successful achievement of sustained local release by siCOL1A2@G5-PBA@Gel.

To further investigate the direct impact of Cy5-siCOL1A2@G5-PBA@Gel gene-drug release on NP cells, NP cells were seeded onto the gel surface (Figure 5C). Following a 2-day induction of inflammation and imbalance in ECM degradation and synthesis by LPS, Western blotting (WB) results revealed (Figure 5D) a significant increase in IL-1β, TNF-α, MMP3, and MMP13 expression in NP cells of the LPS + Gel group compared to the Gel group. Conversely, in the LPS + siCOL1A2@G5-PBA@Gel group, there was a significant decrease in IL-1β, TNF-α, MMP3, and MMP13 expression in NP cells compared to the LPS + Gel group.

In summary, these findings demonstrate that siCOL1A2@G5-PBA@Gel effectively suppresses the inflammatory response and restores the balance of ECM degradation and synthesis, thereby delaying the degeneration of NP cells.

### 3.6. Acupuncture Combined with siCOL1A2@G5-PBA@Gel for Promoting Repair and Regeneration of IVDD in Rats

Based on the findings above, we have determined that the hydrogel system siCOL1A2@G5-PBA@Gel, containing gene therapy, possesses non-toxic, favorable cell compatibility and low hemolysis rates, rendering it suitable for in vivo medical applications. Furthermore, results from in vitro experiments demonstrate the significant capacity of siCOL1A2@G5-PBA@Gel to suppress inflammation and degeneration in NP cells.

**Figure 6:**
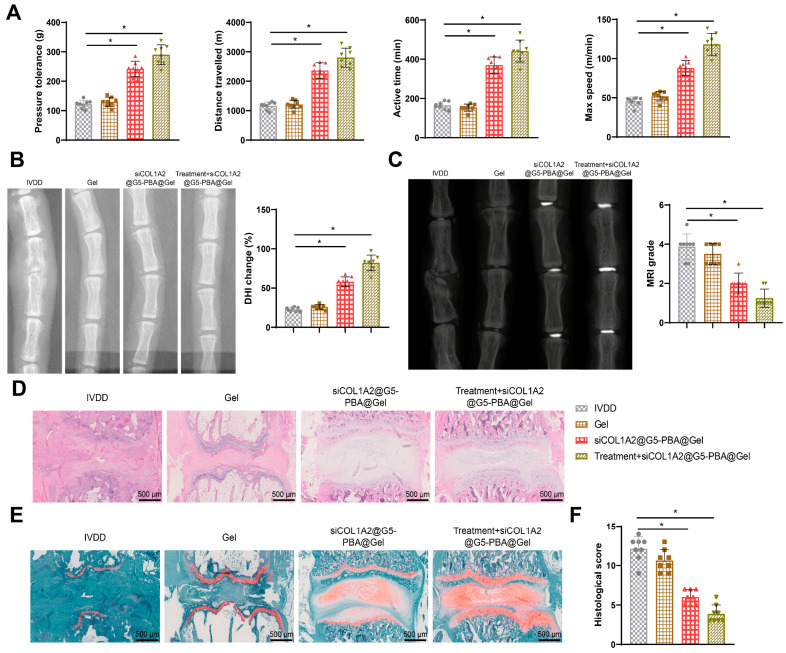
**The impact of siCOL1A2@G5-PBA@Gel joint acupuncture treatment on IVD repair and regeneration in rats.** Note: (**A**) Results of pain response behavioral tests in rats; (**B**) Representative X-ray images of rat intervertebral discs and statistical values of DHI changes; (**C**) Representative quantitative MRI scores of rat intervertebral discs; (**D**) H&E staining of rat intervertebral discs in different treatment groups (scale bar = 500 μm); (**E**) Tomato red O-alcian blue staining of rat intervertebral discs in different treatment groups, with green representing bone tissue and red representing cartilage tissue (scale bar = 500 μm); (**F**) Histological scores of intervertebral discs in each group. N = 8, * indicates *p* < 0.05 when compared between the two groups.

Therefore, we further investigated the synergistic effects of the hydrogel system siCOL1A2@G5-PBA@Gel and acupuncture therapy in vivo. The results revealed that compared to the IVDD group and the Gel treatment group, rats with IVDD in the siCOL1A2@G5-PBA@Gel group exhibited a significant increase in these parameters. Furthermore, the increase was even more pronounced in the acupuncture + siCOL1A2@G5-PBA@Gel combination therapy group (Figure 6A).

The X-ray examination results (Figure 6B) revealed that compared to the IVDD group and the Gel treatment group, rats with IVDD in the siCOL1A2@G5-PBA@Gel group exhibited a significant restoration of intervertebral disc height. Moreover, the restorative effect was even more enhanced in the acupuncture + siCOL1A2@G5-PBA@Gel combination therapy group.

MRI further demonstrated that treatment with siCOL1A2@G5-PBA@Gel promotes the regeneration of degenerated intervertebral discs in rats, slowing down the degenerative process. Additionally, it restores the water content of the intervertebral discs in rats to normal levels, with even better outcomes observed in the combined treatment group (Figure 6C).

Histological examination of rat intervertebral disc tissue sections stained with H&E and Safranin O-Fast Green revealed that, compared to the IVDD group and the Gel treatment group, the IVDD rats in the siCOL1A2@G5-PBA@Gel treatment group showed improvement in disc tissue degeneration and structural damage. There was a clear demarcation between the nucleus pulposus and the annulus fibrosus, reduced loss of cartilaginous components in the disc tissue, with even better restorative effects observed in the combination therapy group (Figure 6D–F).

### 3.7. Acupuncture Combined with siCOL1A2@G5-PBA@Gel Treatment Inhibits Inflammation and Cell Apoptosis in Rats with IVDD

**Figure 7:**
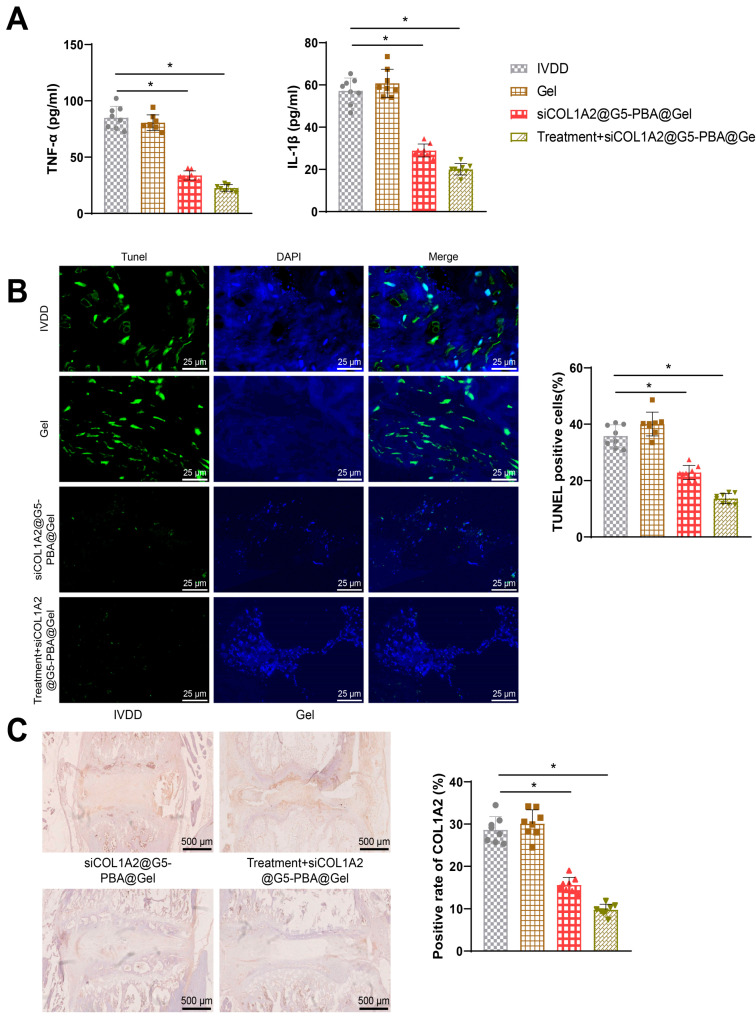
**The impact of siCOL1A2@G5-PBA@Gel combined with acupuncture treatment on inflammation and apoptosis in rat IVDs.** Note: (**A**) ELISA detection of the expression of inflammatory factors TNF-α and IL-1β in rat serum; (**B**) TUNEL staining to evaluate cell apoptosis in rat NP tissue (scale bar = 25 μm); (**C**) Immunohistochemical analysis of COL1A2 protein expression in NP tissues of each group (scale bar = 500 μm). N = 8, * indicates *p* < 0.05 when compared between the two groups.

Further investigation into the impact of acupuncture combined with siCOL1A2@G5-PBA@Gel treatment on inflammation and cell apoptosis in rats with IVDD was conducted. ELISA results revealed that post-acupuncture treatment, the expression of inflammatory factors in rat serum decreased, leading to a reduction in the inflammatory response. Rats treated with a combination of acupuncture and siCOL1A2@G5-PBA@Gel exhibited lower expression of inflammatory factors in serum and a weaker inflammatory response (Figure 7A).

Moreover, TUNEL assay results demonstrated a significant reduction in cell apoptosis in the NP tissue of rats following siCOL1A2@G5-PBA@Gel treatment. Furthermore, the combination therapy resulted in even lower rates of cell apoptosis in the NP tissue of rats (Figure 7B).

Immunohistochemical analysis of COL1A2 protein expression in NP tissues of all groups revealed that post-siCOL1A2@G5-PBA@Gel treatment, the protein expression of COL1A2 significantly decreased in rat NP tissues, with even lower levels in the combination group (Figure 7C).

Based on these comprehensive evaluations, it can be concluded that the siCOL1A2 hydrogel siCOL1A2@G5-PBA@Gel effectively alleviates the progression of IVDD in rats, enhancing the biological function of intervertebral discs. The combined treatment with acupuncture shows superior effects post-treatment, demonstrating potential in the treatment of IVDD.

## 4. Discussion

IVDD is a common spinal condition, and the search for new treatment methods has become crucial due to the limitations of existing therapies [54,55,56]. Traditional approaches such as medication and physical therapy often only provide temporary pain relief and alleviate symptoms without offering a cure [54,55,57]. Therefore, this study aims to investigate the effects and mechanisms of cryogel/hydrogel combined with acupuncture for the treatment of IVDD.

In this study, RNA-seq chip technology was employed to screen key genes related to inflammation, providing a new perspective on the occurrence and progression of the disease [58,59,60]. By using the gene delivery vector G5-PBA, we successfully constructed siCOL1A2@G5-PBA@Gel with high transfection efficiency, which stably binds to the COL1A2 gene and achieves controlled drug release.

In comparison to previous studies, this research has some distinctive features in terms of animal models and evaluation methods. We established a rat model of IVDD and assessed the treatment effects using multiple methods, including pain response behavior, X-ray and MRI imaging examinations, tissue staining, and detection [22,23,24]. These evaluation methods comprehensively assess the impact of treatment on patients and contribute to a better understanding of the treatment’s effects and mechanisms [54,55,57].

The screening method and design of the gene delivery vector in this study provide a theoretical basis for new approaches to the treatment of IVDD. By employing an RNA-seq chip to screen genes related to inflammation, we selected the key gene COL1A2 as an intervention target, which may offer a more targeted treatment plan for patients [61].

The effectiveness of siCOL1A2@G5-PBA@Gel combined with acupuncture treatment was demonstrated through in vivo and in vitro experiments. We found that siCOL1A2@G5-PBA@Gel can inhibit inflammatory reactions, restore cellular metabolic balance, and, therefore, delay cell degeneration. Experimental results show that siCOL1A2-loaded hydrogel (siCOL1A2@G5-PBA@Gel) effectively alleviates the progression of IVDD in rats and improves the biological function of intervertebral discs. Additionally, the combined application demonstrates superior efficacy compared to acupuncture alone [62,63,64].

By comprehensive evaluation of the effects of cryogel/hydrogel combined with acupuncture treatment on a rat model of IVDD, the following conclusions were drawn: Firstly, the rat model of lumbar intervertebral disc protrusion successfully established using mechanical and enzymatic methods exhibits pathological characteristics highly consistent with clinical manifestations of human lumbar intervertebral disc protrusion, providing an effective experimental platform for research. Secondly, the synthesized cryogel/hydrogel demonstrates an ideal porous structure and water absorption capacity, indicating its suitability for mimicking the intervertebral disc environment. Moreover, these materials exhibit good biocompatibility with different cell lines, particularly in promoting normal proliferation and differentiation of nerve cells and MSCs. In the inflammation model, cryogel/hydrogel shows significant anti-inflammatory properties, reducing the expression levels of TNF-α and IL-6. Most importantly, in the animal model, the combined treatment of cryogel/hydrogel acupuncture significantly alleviates IVDD, promotes structural recovery, and facilitates neuronal fiber regeneration in the intervertebral disc region.

Acupuncture is a distinctive Chinese therapeutic method used to treat diseases, employing the medical practice of “treating internal diseases externally”. It involves the application of specific manipulation techniques through the conduction of meridians and acupoints to address systemic illnesses [65]. Currently, in the research on intervertebral disc disease (IVDD) treatment, acupuncture has emerged as a significant therapeutic approach. Studies by Ayne Murata Hayashi et al. have shown that the combination of acupuncture with conventional Western medicine is effective in dogs with thoracolumbar disc disease, reducing the recovery time for mobility and deep pain perception compared to using Western medicine alone [66]. Similar conclusions were also drawn by Hyun-Jung Han et al. [67]. Additionally, Keum Hwa Choi et al. discovered that acupuncture can effectively treat feline multifocal intervertebral disc disease [68]. Nevertheless, the potential application value of acupuncture in the clinical treatment of IVDD still lacks comprehensive examination. This study aimed to explore acupuncture as a complementary treatment approach and found that acupuncture therapy indeed effectively alleviates symptoms in rats with IVDD.

The scientific and clinical value of this study lies in exploring a new treatment approach, specifically cryogel/hydrogel combined with acupuncture, to improve the efficacy of chronic pain in a rat model of IVDD. Traditional treatment methods have limited effectiveness for this condition, highlighting the importance of finding new therapies to alleviate pain and functional impairments in patients. Through the integration of bioinformatics analysis, rat experiments, and in vitro tests, this research demonstrates a significant effect of siCOL1A2@G5-PBA@Gel combined with acupuncture, improving pain and slowing the progression of IVDD.

However, there are several limitations in the present study that need to be addressed. Firstly, the study utilized a rat model, which may differ from the human condition. Therefore, further clinical research is necessary to validate the efficacy and safety of this treatment approach in humans. Currently, the suitability of animal models for understanding the mechanisms and exploring treatments for IVDD depends largely on the correlation and similarity between animal models and human lumbar IVDD. The preparation of IVDD models has a history of nearly 80 years, utilizing experimental animals such as rats, mice, guinea pigs, rabbits, dogs, pigs, sheep, and primates. Due to differences between the spinal structures of experimental animals and humans in terms of growth, anatomy, biomechanics, and biochemistry, as well as variations in the incidence of IVDD with age, an ideal animal model is yet to be universally accepted. Primates, being more closely related to humans, are considered the most ideal model animals; however, their scarcity, challenging maintenance, ethical concerns, and limited suitability hinder widespread use. Sheep exhibit significant anatomical and biomechanical differences in the spine and intervertebral discs compared to humans. Dogs show similarities to humans as their nucleus pulposus cells are replaced by chondrocyte-like cells, yet they have two more lumbar intervertebral discs than humans and thicker cartilaginous endplates. While the internal pressures of intervertebral discs in small quadruped animals like mice, rats, and rabbits may be similar to humans, considering factors such as experimental costs, these animals remain the preferred choice for most researchers. Subsequently, based on practical considerations, we will strive to adopt more relevant animal models before embarking on clinical investigations. Secondly, the inhibitory effect of siCOL1A2@G5-PBA@Gel on inflammation has only been demonstrated through in vitro experiments. Hence, more in-depth mechanistic studies are required to explore its mode of action. Additionally, this study did not comprehensively analyze the treatment mechanism, leaving room for future investigations.

**Figure 8:**
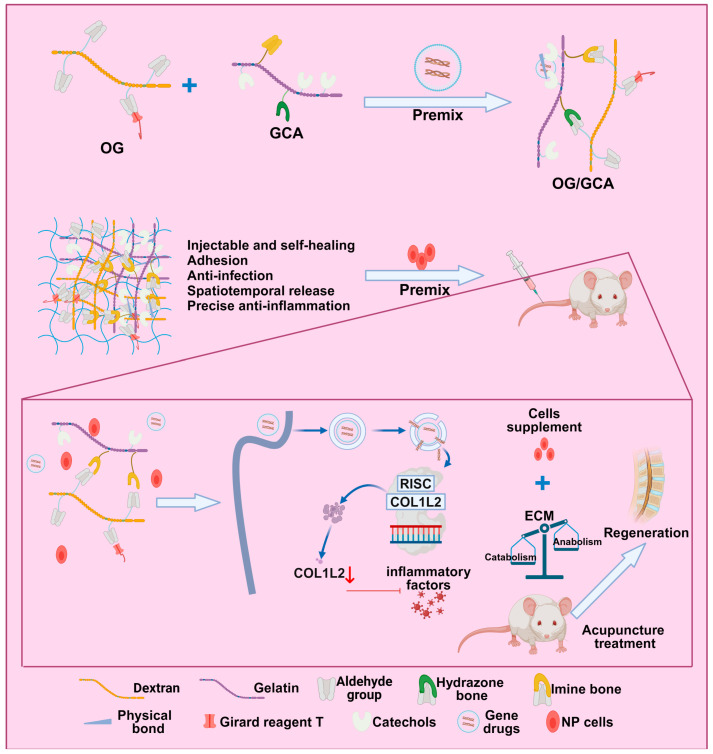
Mechanism diagram of cryogel/hydrogel combined acupuncture treatment for improving IVDD.

Looking ahead, based on the findings of this study, clinical trials can be conducted to verify the effectiveness and safety of siCOL1A2@G5-PBA@Gel combined with acupuncture in patients with IVDD, thereby confirming its clinical value. Furthermore, further research can examine the effects of this treatment method on improving patients’ mobility impairments and quality of life, offering a better treatment option for individuals with spinal disorders. Additionally, a thorough investigation of the treatment mechanism can elucidate how siCOL1A2@G5-PBA@Gel combined with acupuncture alleviates the progression of lumbar IVDD by improving inflammatory response and restoring cellular metabolic balance, providing a theoretical basis for the development of more effective treatment strategies (Figure 8).

## Data Availability

All data can be provided as needed.

## Supplementary Materials

The following supporting information can be downloaded at: https://www.mdpi.com/article/10.3390/bioengineering11111066/s1. Figure S1. Screening target genes regulating inflammation in IVDD. Figure S2. PPI network analysis of key targets in inflammation-regulated IVDD. Figure S3. Further selection of hub genes using machine learning. Figure S4. Successful construction of siCOL1A2@G5-PBA complex. Figure S5. Characterization of OG/GCA hydrogel.
