# Supplementary material for: Regenerative Outcomes of Combining siCOL1A2 Hydrogel with Acupuncture in a Rat Model of Chronic Intervertebral Disc Degeneration
Source: Bioengineering (Basel). 2024 Oct 25;11(11):1066. doi: 10.3390/bioengineering11111066 (PMC11591507; doi:10.3390/bioengineering11111066)
Supplement: Supplementary file 1 [file bioengineering-11-01066-s001.zip › bioengineering-3086982-supplementary.pdf]

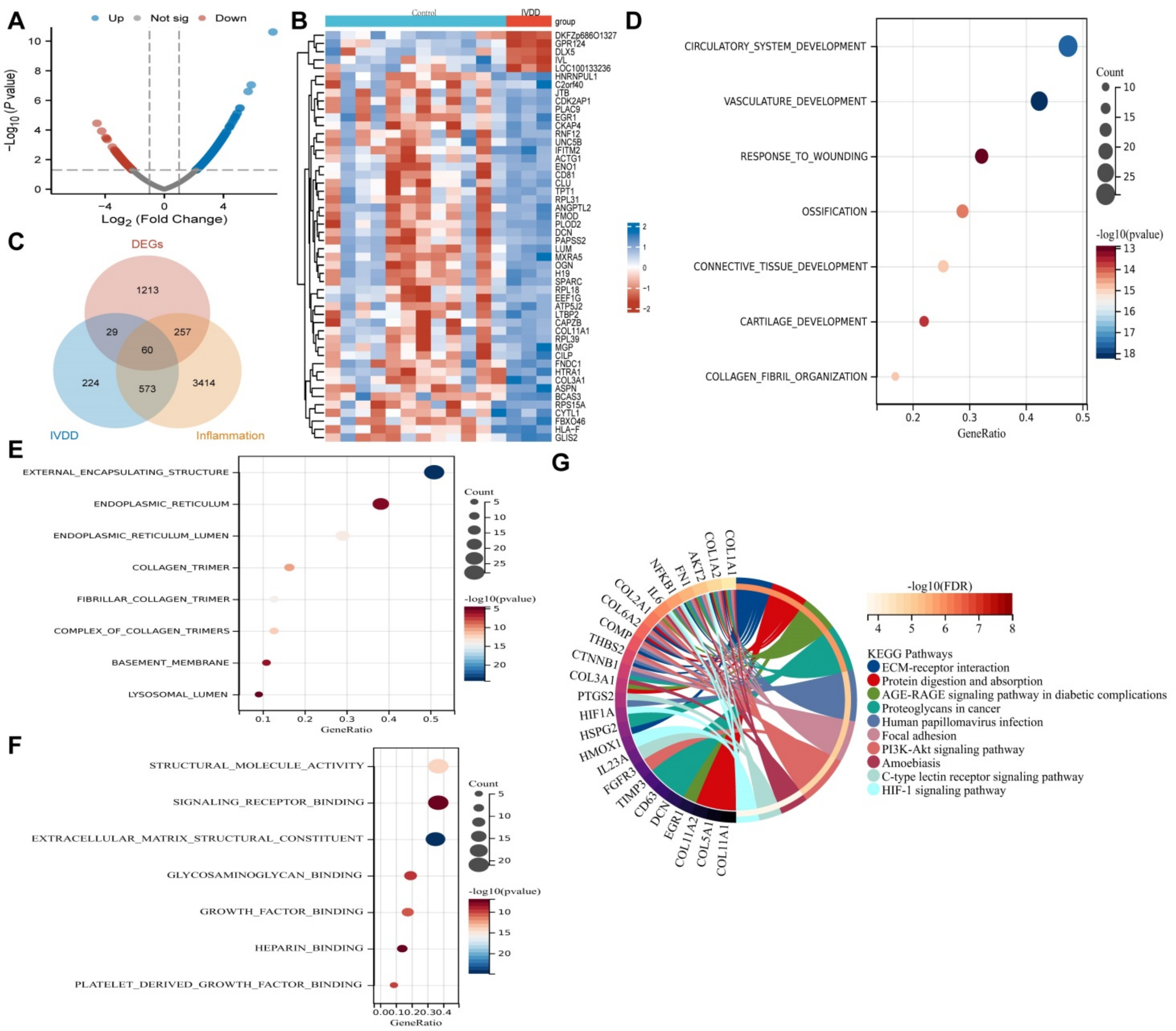

Figure S1. Screening target genes regulating inflammation in IVDD.

Note: (A) The volcano plot of differential gene expression in the GSE15227 dataset indicates upregulated genes in blue, downregulated genes in red, and genes with no significant difference in gray (IVDD: n=3; Control: n=12); (B) The heatmap demonstrates the expression patterns of genes in IVDD and control groups. The depth of color signifies the level of gene expression, with red indicating low expression and blue indicating high expression; (C) Intersection Venn diagram showing the DEGs from GSE15227 dataset and genes related to IVDD and inflammation in the GeneCards database; (D-F) Bubble plots of GO functional analysis of target genes regulating inflammation in IVDD at the BP level (E), CC level (F), and MF level (G), where the size of the dots represents the number of selected genes, and the colors indicate the P-values of enrichment analysis; (G) Circular plot of KEGG pathway enrichment analysis for target genes regulating

inflammation in IVDD.

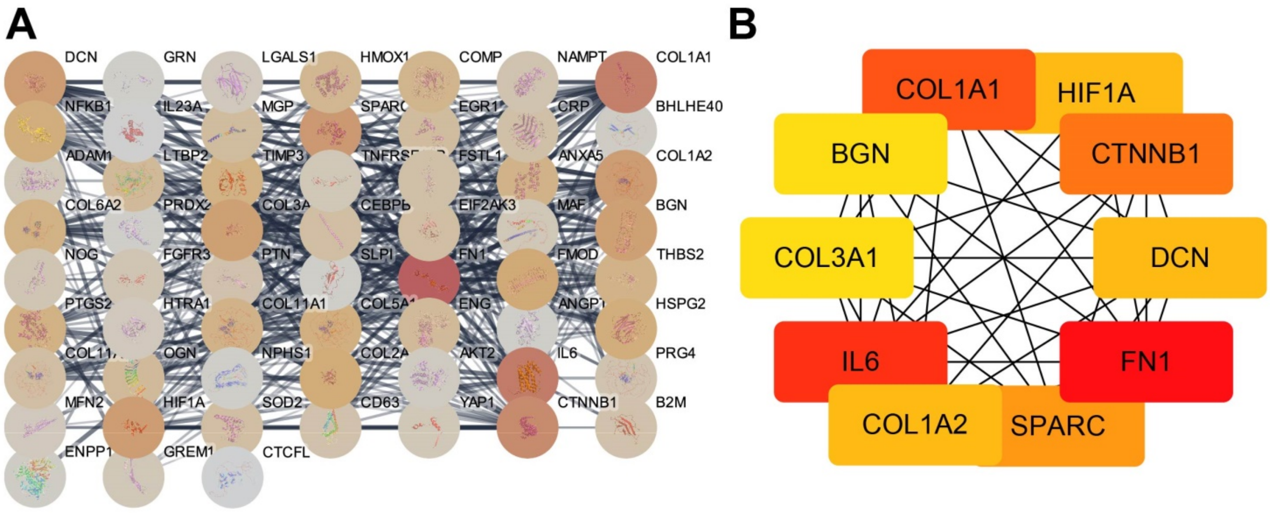

Figure S2. PPI network analysis of key targets in inflammation-regulated IVDD.

Note: (A) The PPI network of 60 candidate genes involved in inflammation-regulated IVDD was constructed by importing the genes into the STRING database to obtain corresponding protein-protein interaction relationships using Cytoscape software. The background color of the genes indicates the Degree value, with darker colors indicating higher values; (B) The top 10 genes ranked by Degree values were calculated using the CytoHubba plugin. The background color of the genes indicates the Degree value, with darker colors indicating higher values.

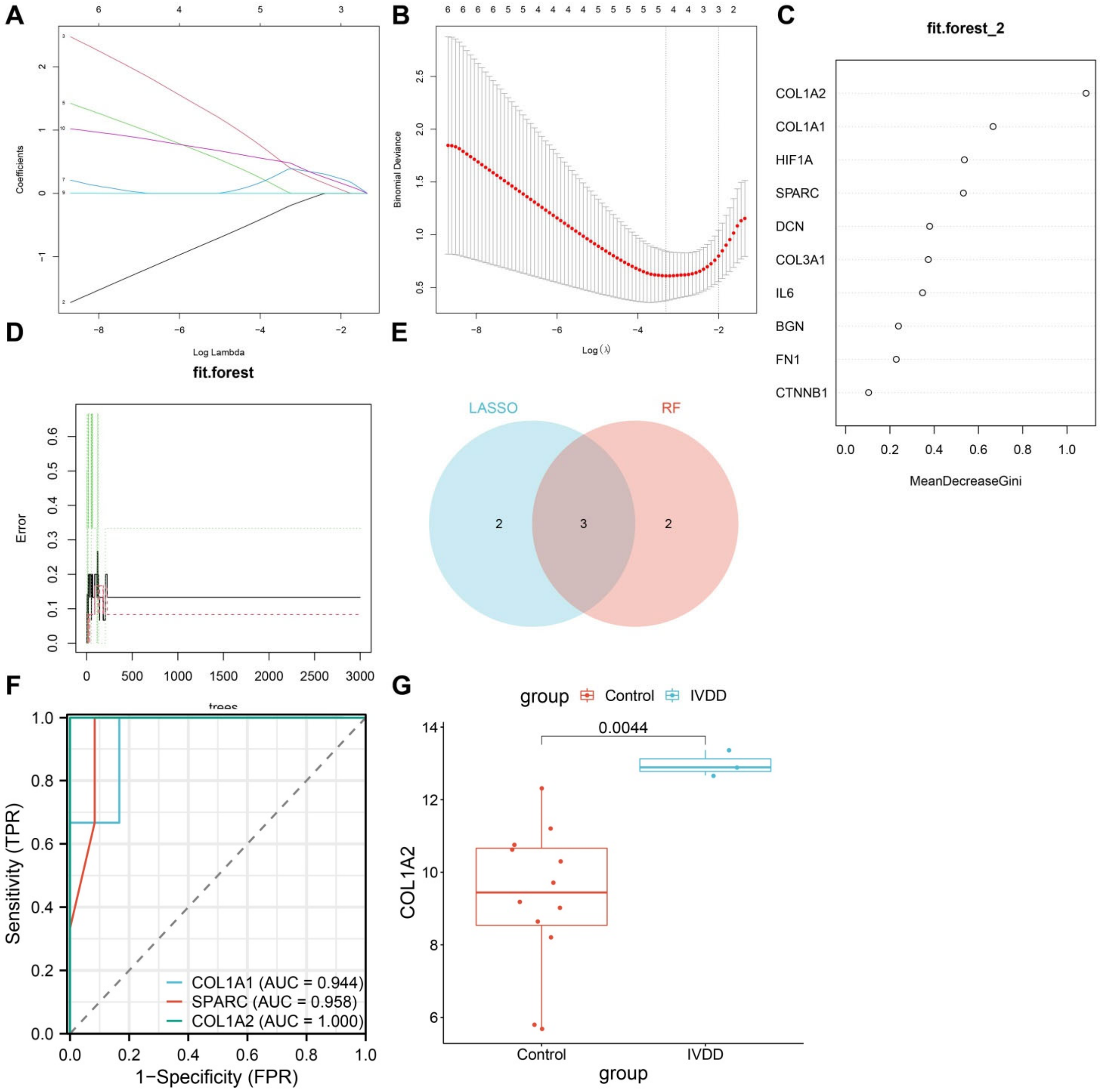

Figure S3. Further selection of hub genes using machine learning.

Note: (A) The coefficients of different genes vary with the λ values; (B) The optimal parameter (lambda) obtained through cross-validation LASSO regression analysis; (C-D) Identification of important features of the 10 genes using the RF algorithm; (E) Venn diagram depicting the overlap of the top 5 genes selected by LASSO and RF; (F) Diagnostic performance of the hub genes in the training dataset GSE15227, shown by the area under the curve (AUC). AUC values close to 1 indicate perfect prediction, values above 0.90 indicate high accuracy, values in the range of 0.70-0.90 indicate moderate accuracy, and AUC values in the range of 0.50-0.70 indicate low accuracy; (G) Boxplots showing the expression levels of hub genes in the GSE15227 dataset,

comparing the control group with IVDD patients. A significance level of $P < 0.05$ was considered statistically significant.

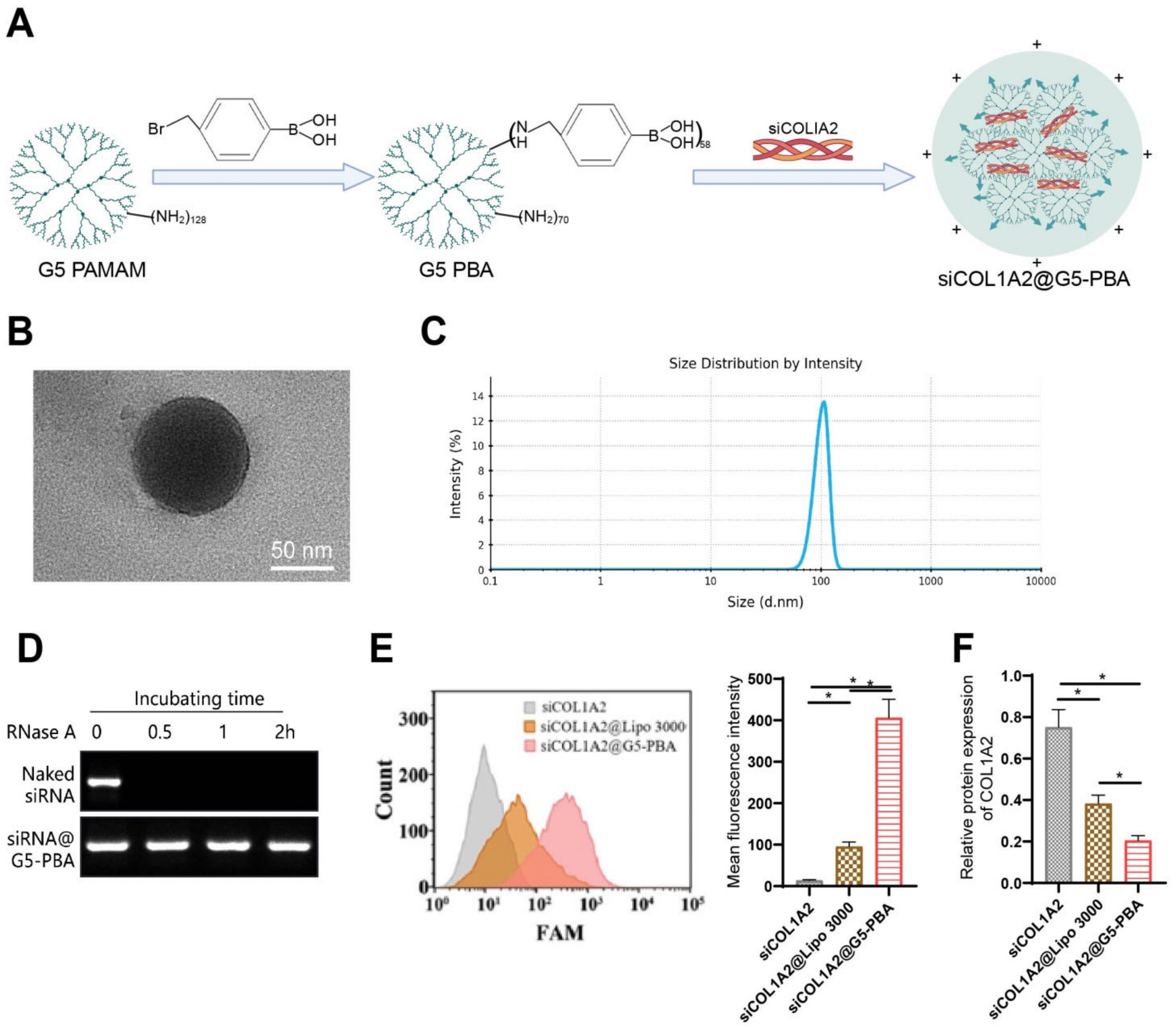

Figure S4. Successful construction of siCOL1A2@G5-PBA complex.

Note: (A) Schematic representation of G5-PBA synthesis and siCOL1A2@G5-PBA complex preparation; (B) TEM image of siCOL1A2@G5-PBA complex (scale bar=50 nm); (C) Size distribution of siCOL1A2@G5-PBA; (D) Agarose gel electrophoresis of the complex after RNase digestion; (E) Flow cytometry analysis of FAM-siRNA@G5-PBA uptake in NP cells; (F) WB detection of COL1A2 protein level in NP cells. * indicates $p < 0.05$. The experiment was repeated 3 times.

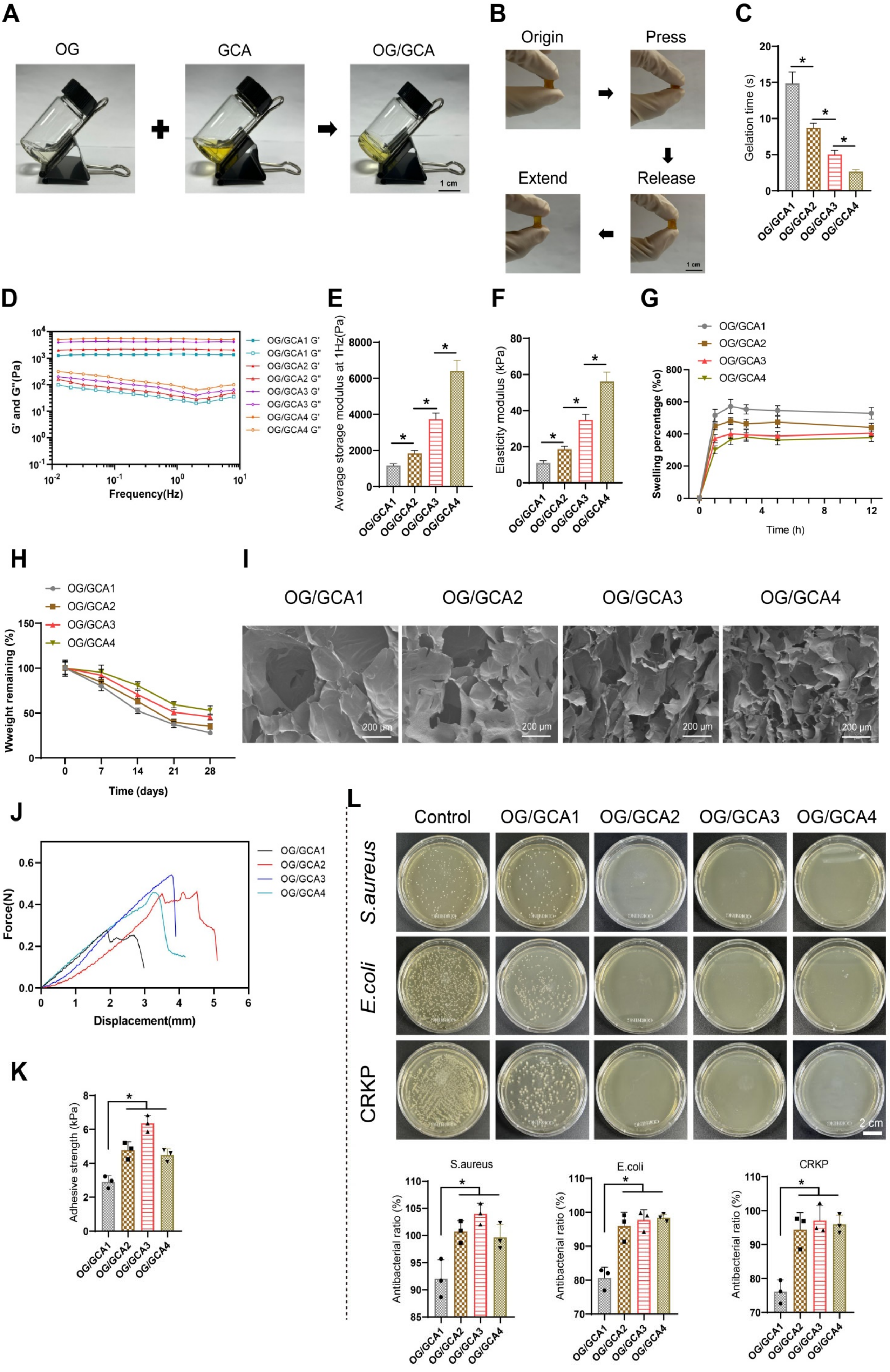
A
OG
GCA
OG/GCA
1 cm
B
Origin
Press
Extend
Release
1 cm
C
Gelation time (s)
OG/GCA1
OG/GCA2
OG/GCA3
OG/GCA4
D
G' and G''(Pa)
Frequency(Hz)
OG/GCA1 G'
OG/GCA1 G''
OG/GCA2 G'
OG/GCA2 G''
OG/GCA3 G'
OG/GCA3 G''
OG/GCA4 G'
OG/GCA4 G''
E
Average storage modulus at 1Hz(Pa)
F
Elasticity modulus (kPa)
G
Swelling percentage (%o)
Time (h)
H
Wweight remaining (%)
Time (days)
I
OG/GCA1
OG/GCA2
OG/GCA3
OG/GCA4
200 μm
J
Force(N)
Displacement(mm)
K
Adhesive strength (kPa)
L
Control
S.aureus
E.coli
CRKP
2 cm
S.aureus
E.coli
CRKP
Antibacterial ratio (%)

Figure S5. Characterization of OG/GCA hydrogel.

Note: (A) Photographs of OG solution, GCA solution, and OG/GCA hydrogel (scale bar=1 cm); (B) Photograph demonstrating the compressibility and extensibility of the hydrogel (scale bar=1 cm); (C) Tilt test of hydrogel coagulation time at different polymer concentrations (n=3); (D) Frequency sweep test of hydrogel at different concentrations at 37°C (from 0.01 to 10 Hz); (E) Quantitative average storage modulus of the hydrogel at 1 Hz determined from the frequency sweep test (n=3); (F) Elastic modulus in the compression curve of the hydrogel in the range of 10-20% strain (n=3); (G) Swelling characteristics of the hydrogel in PBS (n=3); (H) *In vitro* degradation performance of the hydrogel measured by oscillating at 100 rpm at 37°C (n=3); (I) SEM image of the hydrogel (scale bar=200 μm). OG/GCA1: 5% OG and 10% GCA; OG/GCA2: 8.5% OG and 10% GCA; OG/GCA3: 8.5% OG and 15% GCA; OG/GCA4: 12% OG and 15% GCA. (J) Typical force-displacement curves of pigskin combined with hydrogels of different concentrations; (K) Calculation of quantitative adhesive strength of hydrogels based on force-displacement curves; (L) Photographs of bacterial colonies formed by Staphylococcus aureus, Escherichia coli, and CRKP after treatment with different hydrogels, scale bar=2 cm. * indicate $p < 0.05$ compared to another group, respectively. The experiment was repeated 3 times.
